# Supplementary material for: Integrative taxonomy clarifies the evolution of a cryptic primate clade
Source: Nat Ecol Evol. 2024 Sep 27;9(1):57–72. doi: 10.1038/s41559-024-02547-w (PMC11726463; doi:10.1038/s41559-024-02547-w)
Supplement: Supplementary file 1 — Supplementary results, discussion, methods and figures. [file 41559_2024_2547_MOESM1_ESM.pdf]

nature ecology & evolution

Supplementary information https://doi.org/10.1038/s41559-024-02547-w

Integrative taxonomy clarifies the evolution of a cryptic primate clade

In the format provided by the authors and unedited

Supplementary Table of contents

Supplementary results and discussion 2

Phylogenetic inference (Supplementary Figs. 1 to 6) 2

Species delimitation and diagnosis (Supplementary Figs. 7 and 8) 9

M. rufus, M. berthae, M. myoxinus 9

M. lehilahytsara, M. mittermeieri 10

M. mamiratra, *M. margotmarshae, M. sambiranensis* 11

M. arnholdi, *M.* sp. 1 12

M. boraha, M. simmonsi 14

M. jollyae, M. marohita, *M. gerpi* 15

M. macarthurii, *M. jonahi* 17

M. manitatra, *M. ganzhorni*, *M. murinus* 18

M. ravelobensis, *M. bongolavensis, M. danfossi* 21

Singletons: *M. tanosi*, *M. tavaratra*, *M. griseorufus* 23

Divergence time estimation (Supplementary Figs. 9 to 14) 25

Biogeographic reconstruction and diversification rate analysis (Supplementary Figs. 15 to 17) 32

Morphological stasis and neutral climatic niche evolution (Supplementary Figs. 18 to 21) 36

Change in conservation status 40

Supplementary methods 41

Sampling (Supplementary Fig. 22) 41

Library preparation 42

Species delimitation (Supplementary Figs. 23 to 27) 42

Genomics (isolation-by-distance): 42

Genomics (genealogical divergence index): 47

Reproductive activity: 48

Acoustic communication: 49

Divergence time estimation 51

Modelling morphological and climatic niche evolution (Supplementary Figs. 28 to 29) 53

References 55

Supplementary results and discussion

Phylogenetic inference (Supplementary Figs. 1 to 6)

We inferred the first comprehensive phylogeny for the genus *Microcebus* under five missing data thresholds and with two complementary algorithms (maximum likelihood, quartet-based). With respect to species-level divergences, maximum likelihood phylogenies are congruent across filtering schemes and ultrafast bootstrap support is high (Supplementary Figs. 1 to 5). Quartet-based inference on a thinned SNP set largely supports the maximum likelihood topology but standard bootstrap values decrease and incongruencies increase with higher amounts of missing data, both for individual- and for species-level assignments (Supplementary Figs. 1 to 6). We therefore used the consistent maximum likelihood topology for downstream analyses (i.e., divergence time estimation, biogeographic reconstruction and the modelling of morphological stasis and climatic niche diversification).

Our topology supports a basal split between the *M. murinus* group, *M. griseorufus* and the clade comprised of *M. bongolavensis*, *M. danfossi* and *M. ravelobensis* on the one hand, and all other *Microcebus* species on the other hand. Among the latter, pairs and triplets of candidate species branch off consecutively, starting with *M. jonahi* and *M. macarthurii* of northeastern Madagascar, followed by *M. gerpi*, *M. jollyae* and *M. marohita* of the central east coast and *M. boraha* and *M. simmonsi* from the areas in between. Subsequently, there is a bifurcation separating the species of northern Madagascar (i.e., *M. arnholdi*, *M.* sp. 1, *M. tavaratra*, *M. mamiratra*, *M. margotmarschae*, *M. sambiranensis*) and the remaining species from the dry central-western and the humid eastern forests of Madagascar (i.e., *M. berthae*, *M. myoxinus*, *M. rufus*, *M. tanosi*, *M. lehilahytsara*, *M. mittermeieri*).

Previous phylogenies for the genus *Microcebus* often relied on a limited set of species and/or genes and exhibited low support or short branch lengths especially at deeper nodes[1–8]. It is beyond the scope of this work to discuss all conflicting phylogenetic hypotheses. Notably, however, the placement of the clade comprising *M. bongolavensis*, *M. danfossi* and *M. ravelobensis* appeared to be particularly difficult to resolve. Our topology places this clade with high support as sister to the *M. murinus* group and *M. griseorufus*, which is in line with Weisrock et al.[6] and a recent analysis of ultra-conserved elements[9]. In contrast, Fauskee et al.[10] suggest that this placement may be an artefact caused by ancient gene flow between the stem of the clade and that of the *M. murinus* group and *M. griseorufus*, and that it rather is sister to the other major clade in the *Microcebus* phylogeny. Further research is necessary to clarify its position and identify the role of gene flow particularly during the early diversification of the genus *Microcebus*.

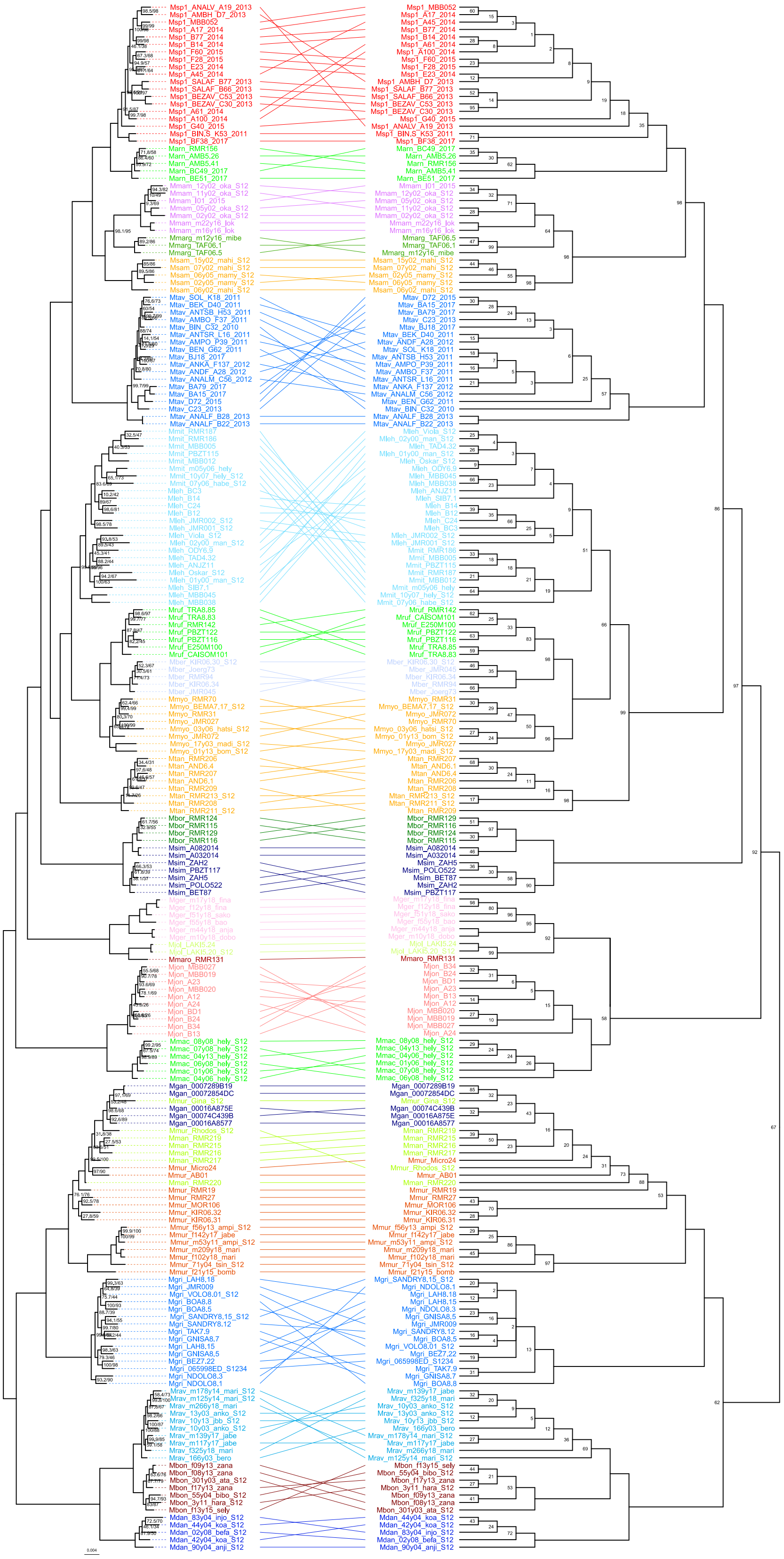

Supplementary Figure 1: Maximum likelihood (left) and quartet-based (right) phylogenies inferred with IQ-TREE and SVDquartets, respectively, from a SNP set with 5% maximum missing data per site. Node labels represent percent SH-aLRT/ultrafast bootstrap support (left) and percent bootstrap support (right) if below 100. Individuals are coloured according to candidate species. Scale is substitutions per site.

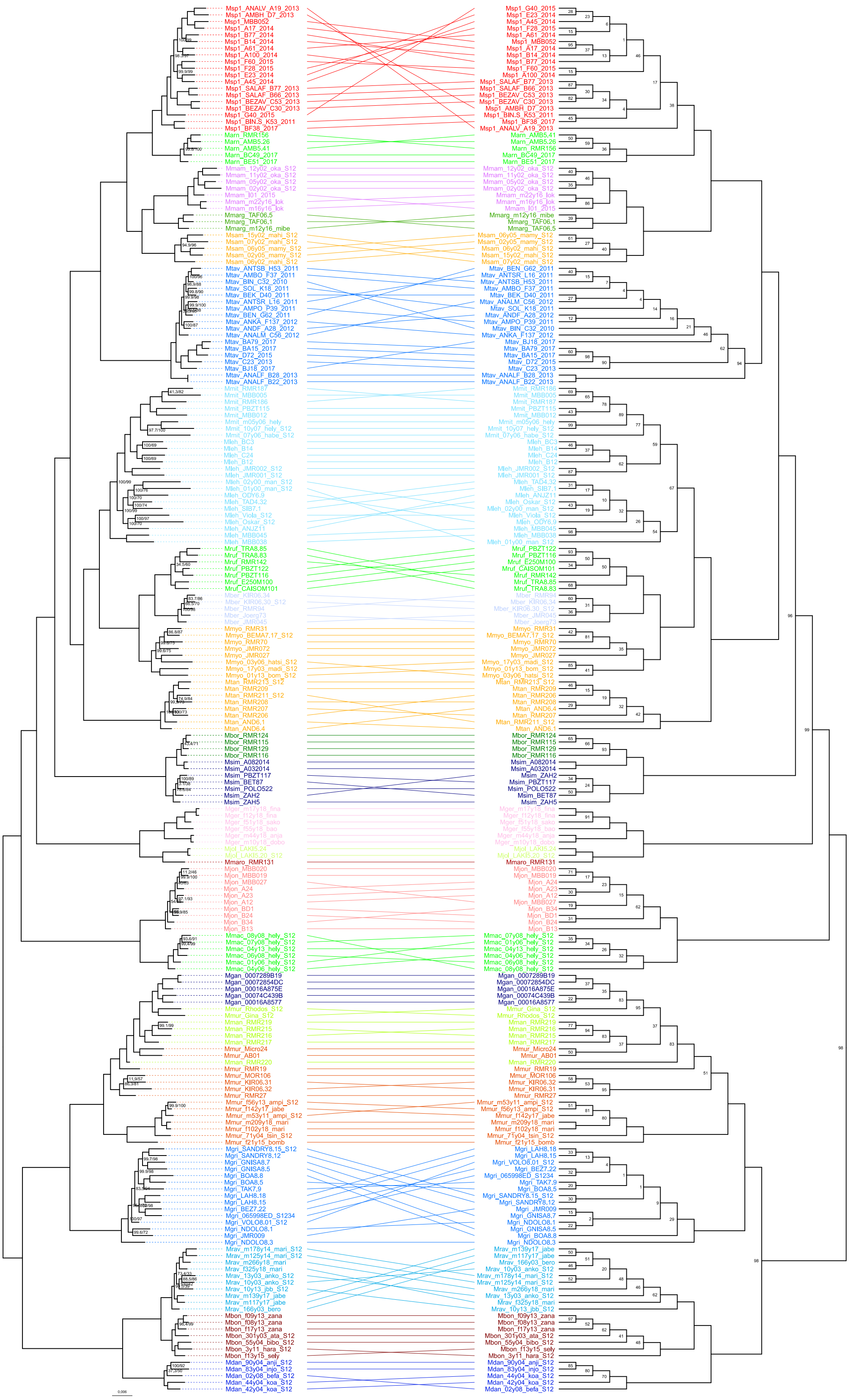

Supplementary Figure 2: Maximum likelihood (left) and quartet-based (right) phylogenies inferred with IQ-TREE and SVDquartets, respectively, from a SNP set with 25% maximum missing data per site. Node labels represent percent SH-aLRT/ultrafast bootstrap support (left) and percent bootstrap support (right) if below 100. Individuals are coloured according to candidate species. Scale is substitutions per site.

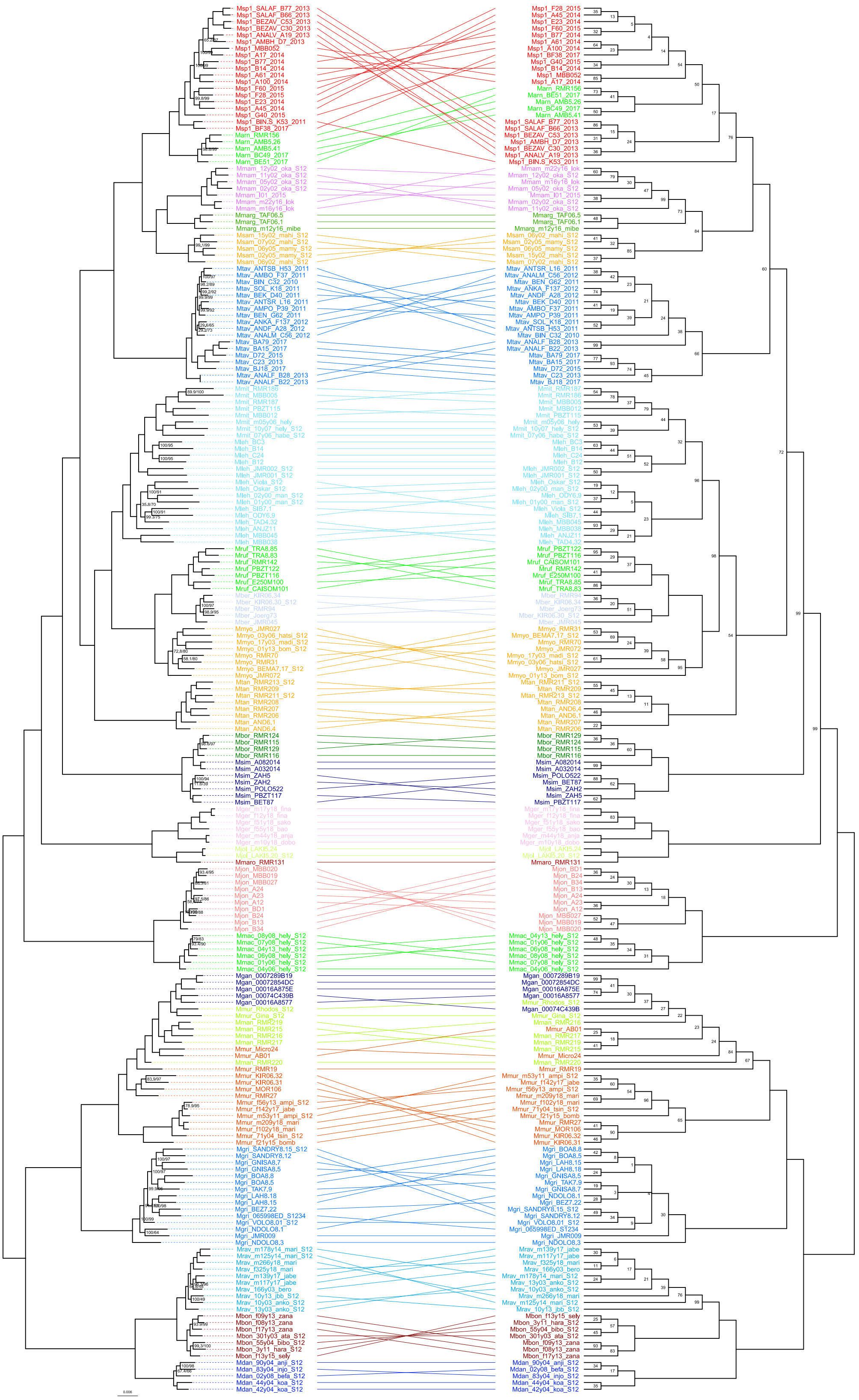

Supplementary Figure 3: Maximum likelihood (left) and quartet-based (right) phylogenies inferred with IQ-TREE and SVDquartets, respectively, from a SNP set with 50% maximum missing data per site. Node labels represent percent SH-aLRT/ultrafast bootstrap support (left) and percent bootstrap support (right) if below 100. Individuals are coloured according to candidate species. Scale is substitutions per site.

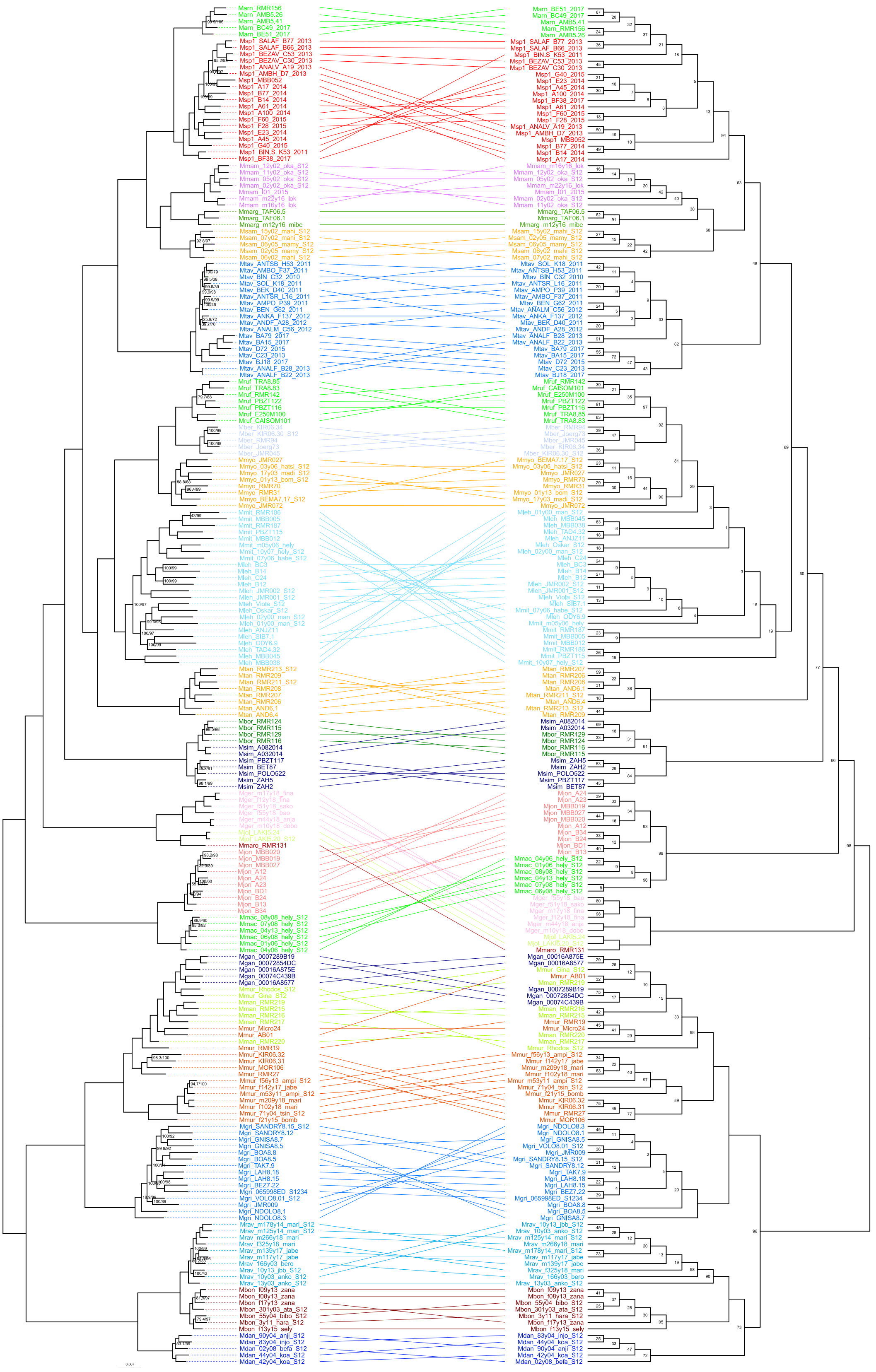

Supplementary Figure 4: Maximum likelihood (left) and quartet-based (right) phylogenies inferred with IQ-TREE and SVDquartets, respectively, from a SNP set with 75% maximum missing data per site. Node labels represent percent SH-aLRT/ultrafast bootstrap support (left) and percent bootstrap support (right) if below 100. Individuals are coloured according to candidate species. Scale is substitutions per site.

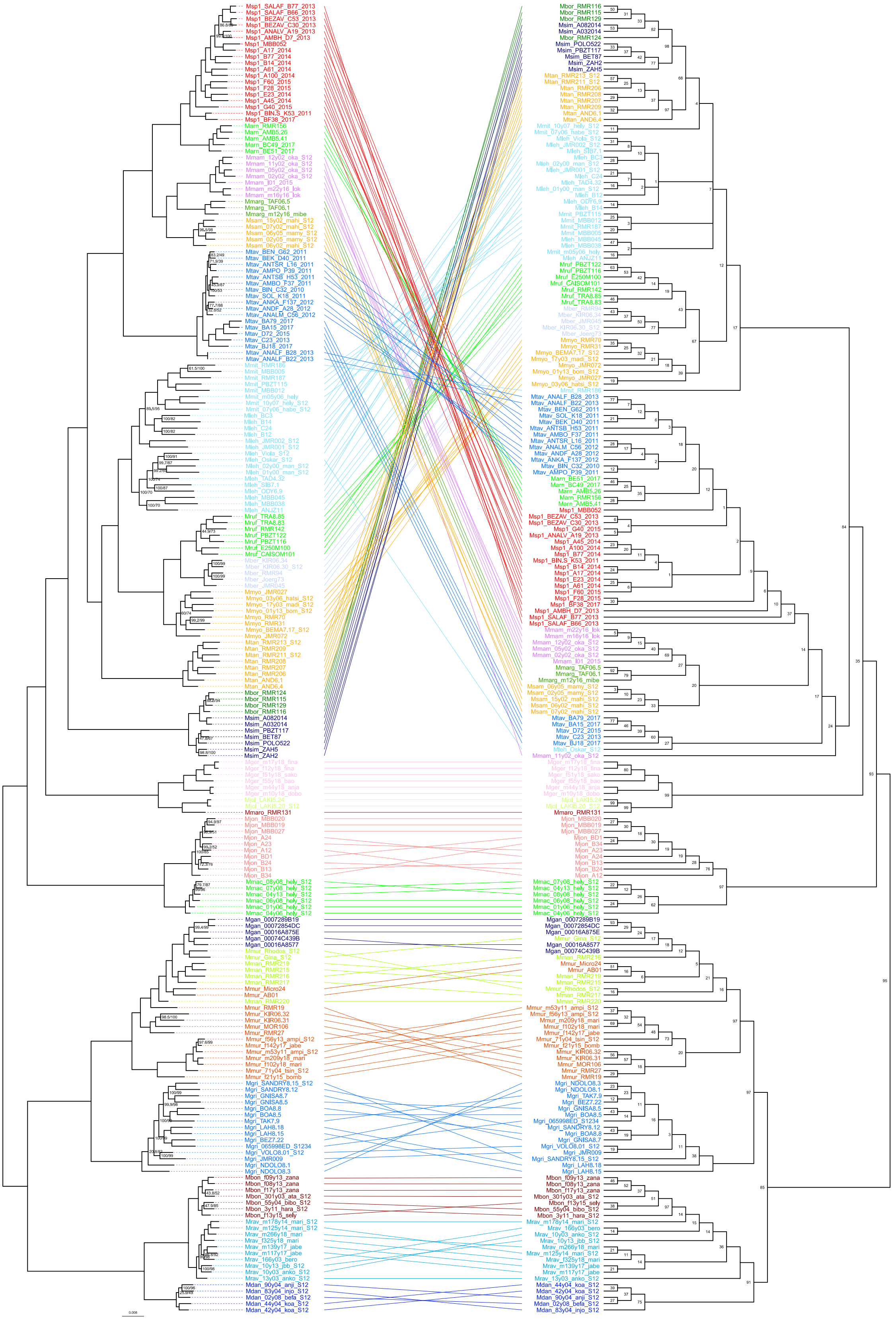

Supplementary Figure 5: Maximum likelihood (left) and quartet-based (right) phylogenies inferred with IQ-TREE and SVDquartets, respectively, from a SNP set with 95% maximum missing data per site. Node labels represent percent SH-aLRT/ultrafast bootstrap support (left) and percent bootstrap support (right) if below 100. Individuals are coloured according to candidate species. Scale is substitutions per site.

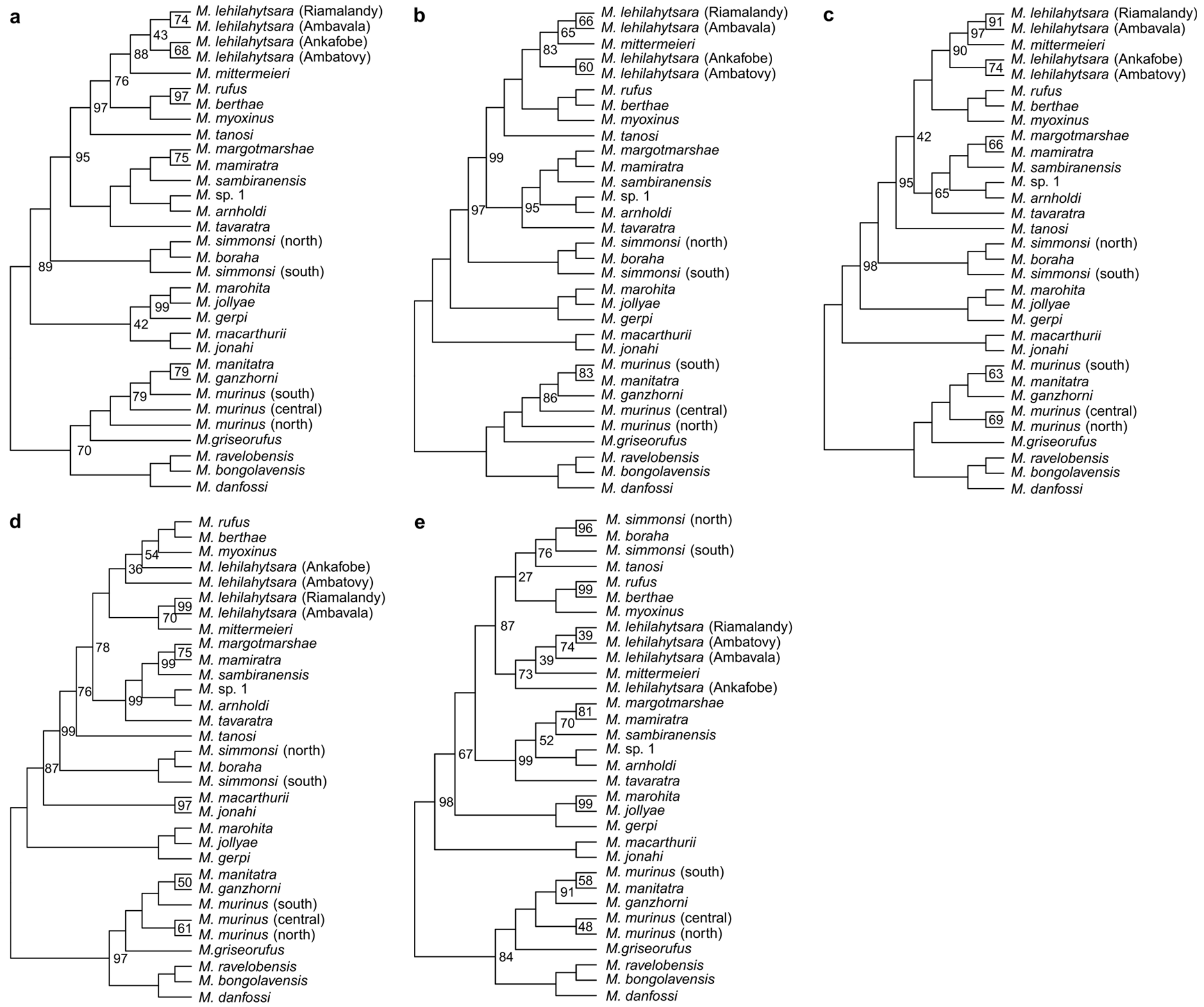

Supplementary Figure 6: Species trees inferred with SVDquartets from SNP sets with 5% (**a**), 25% (**b**), 50% (**c**), 75% (**d**) and 95% (**e**) maximum missing data per site. Node labels represent percent bootstrap support if below 100.

Species delimitation and diagnosis (Supplementary Figs. 7 and 8)

The following sections detail delimitation results for each group of candidate species. A summary of these results is given in Supplementary Table 1.

M. rufus (Geoffroy, 1834)*, M. berthae* (Rasoloarison et al., 2000)*, M. myoxinus* (Peters, 1852):

M. rufus and M. myoxinus are the earliest recognized *Microcebus* species after *M. murinus* and were described based on differences in coloration and/or morphology[11,12]. *M. berthae* was described from Kirindy Private Reserve (PR) due to differentiation in external morphological, cranial and dental measurements (three individuals) as well as mtDNA (four individuals) compared to other *Microcebus* species in western Madagascar[1,13].

The three species occur allopatrically, with *M. rufus* inhabiting montane humid forests on the east coast with two population strongholds in Ranomafana National Park (NP) and Andringitra NP (Extended Data Fig. 2a). The other two species occur in the dry forests of western Madagascar (*M. berthae* in Menabe Antimena Protected Area [PA]; *M. myoxinus* between the rivers Tsiribihina and Betsiboka and inside Tsingy de Bemaraha NP).

Patterns of isolation-by-distance (IBD) in genomic data are inconclusive for delimitation of the three candidates, as neither the intra- nor the interspecific model are clearly rejected (Extended Data Fig. 2d; Supplementary Table 2). However, interspecific genetic distances are slightly higher than intraspecific ones when considering similar geographic distances (Extended Data Fig. 2d). Furthermore, our analyses indicate that the candidates are reciprocally monophyletic (Extended Data Fig. 2b), present distinct genomic clusters (Extended Data Fig. 2c) and have intermediate genealogical divergence indices (*gdi*) (Extended Data Fig. 2e; Supplementary Table 3). Morphometric data are not concordant with an intraspecific model of IBD and reveal major differentiation among candidates, with low hypervolume overlap (Extended Data Fig. 2f, Supplementary Tables 4 and 5). Similarly, climatic niche overlap (Schoener's D) is particularly low and significantly different from a null distribution (Extended Data Fig. 2g; Supplementary Table 6), as can be expected given the disjunct distributions of the three taxa. We also observe a later onset of oestrus in female *M. berthae* compared to *M. myoxinus* and *M. rufus* (Extended Data Fig. 2h), indicating that there are sSupplementary Table differences in female seasonal reproductive activation between these species. Since we do not find any evidence for ongoing gene flow between the three candidates but detect differentiation in morphometry, climatic niche and reproductive activity, our findings support the current taxonomic classification of the candidates as distinct species. Notably, genetic samples are currently lacking for the northern part of the distribution of *M. rufus*, which would shed further light on the low genetic differentiation between *M. rufus* and

M. berthae (Extended Data Fig. 2de). It is unlikely, however, that additional sampling will challenge our general conclusion given the clear differentiation of the three candidates across multiple lines of evidence, our extensive sampling covering large parts of their known distributions and the disjunct ranges of *M. berthae* and *M. rufus* (Extended Data Fig. 2a; Supplementary Tables 13 to 16).

Based on our sampling and genetic analyses, we do not identify separate evolutionarily significant units other than these candidates (Extended Data Fig. 2b). While all three species occur in protected areas, these safeguards may not prevent them from extinction, as suspected for *M. berthae*[14].

M. lehilahytsara (Roos & Kappeler, 2005)*, M. mittermeieri* (Louis et al., 2006):

The initial descriptions of these two candidates were based on the northern and southern extreme points of their known combined distribution (Extended Data Fig. 3a). *M. lehilahytsara* was described from Andasibe based on differentiation in the mitochondrial cytochrome b gene of two individuals to other previously described *Microcebus* species[15]. The description of *M. mittermeieri* was based on molecular diagnosability in the mitochondrial D-loop region of six individuals at Anjanaharibe-Sud Special Reserve (SR)[2]. However, at the time of description, sequences of *M. mittermeieri* were not compared to *M. lehilahytsara*. The population genomics and morphometric diversity of these two species have recently been studied using extensive data at a wide range of sampling sites[16–18], providing convincing evidence that they are not valid species but exhibit intraspecific geographic variation in genomic and morphological diversity. Accordingly, these candidates should be considered a single species that is distributed along the humid northeastern coast of Madagascar with differentiated populations in isolated forest fragments of the central highlands. The southern and northern distributional limits of this species are the rivers Mangoro and Bemarivo, respectively, making it the *Microcebus* species with the second-largest distribution after *M. murinus*.

Our analyses support these findings, as patterns of genomic IBD between candidates appear to be an extension of within-candidate patterns, in line with an intraspecific model of diversification (Extended Data Fig. 3d, Supplementary Table 2). This is also supported by the fact that *M. mittermeieri* is phylogenetically nested in *M. lehilahytsara* (Extended Data Fig. 3b), with strong evidence for ongoing or recent gene flow among populations (Extended Data Fig. 3c) and particularly low *gdi* values (Extended Data Fig. 3e, Supplementary Table 3). Furthermore, we find comparably high hypervolume overlap in morphometry (no significant pattern of IBD is detected; Extended Data Fig. 3f, Supplementary Tables 4 and 5) and intermediate to high overlap in climatic niches, not deviating significantly from the null

distribution (Schoener's D; Extended Data Fig. 3g, Supplementary Table 6). Finally, reproductive schedules indicate an overlap and provide no evidence for differentiation (Extended Data Fig. 3h). However, average monthly sample size is low for *M. mittermeieri* (n=5) and *M. lehilahytsara* (n=9) (Supplementary Table 16), and there may be variation in reproductive activity among populations of the widely distributed species *M. lehilahytsara* due to environmental plasticity[17,19].

The detailed population genomic analysis in Tiley et al.[18] suggests that this taxon is composed of at least six genetically differentiated, reciprocally monophyletic lineages that deserve conservation attention, i.e., a northern humid forest lineage (previous *M. mittermeieri*), two central humid forest lineages (at Ambavala/Madera and at Riamalandy SR, respectively), a southern humid forest lineage (at Andasibe-Mantadia NP and Tsinjoarivo-Ambalaomby New Protected Area [NPA]) and two Central Highland populations at Ankafobe unprotected forest and Ambohitantely SR, respectively (Extended Data Fig. 3b; note that the lineage at Ambohitantely SR is not represented due to low sample quality).

M. mamiratra (Andriantompohavana et al., 2006), *M. margotmarshae* (Louis et al., 2008), *M. sambiranensis* (Rasoloarison et al., 2000):

M. sambiranensis was described from Manongarivo SR based on differentiation in external morphological measurements (six individuals) and mtDNA (three individuals) to other *Microcebus* species in western Madagascar[13]. The descriptions of *M. mamiratra* and *M. margotmarshae* were based on molecular diagnosability in mitochondrial D-loop and PAST sequence fragments of four and five individuals at Lokobe SR and Antafondro Classified Forest SR, respectively, compared to previously described *Microcebus* species (including *M. sambiranensis*)[3,20].

The three candidates are distributed in dry deciduous and transitional forests of the Sambirano region of northwestern Madagascar. Their distributions are separated by large rivers: The Sambirano River separates *M. mamiratra* from *M. margotmarshae*, and the Andranomalaza River separates *M. margotmarshae* from *M. sambiranensis* (Extended Data Fig. 4a).

Patterns of IBD in genomic data are inconclusive for the delimitation of the three candidates, as neither the intra- nor the interspecific model were clearly rejected (Extended Data Fig. 4d, Supplementary Table 2). However, interspecific genetic distances are slightly higher than intraspecific ones, when considering similar geographic distances (Extended Data Fig. 4d). Furthermore, our analyses indicate that the candidates are reciprocally monophyletic (Extended Data Fig. 4b) and form distinct genomic clusters (Extended Data Fig. 4c).

Genealogical divergence is intermediate (Extended Data Fig. 4e, Supplementary Table 3). While overlap in climatic niches (Schoener's D) is high among the candidates and does not deviate significantly from a null distribution (as can be expected given the proximity of their distributions; Extended Data Fig. 4g, Supplementary Table 6), hypervolume overlap in morphometry is particularly low and morphometric data are not concordant with an intraspecific model of IBD (Extended Data Fig. 4f, Supplementary Tables 4 and 5). Data on reproductive activity do not allow the detection of differences in reproductive schedules between candidates because there is only limited overlap in assessed months and average monthly sample sizes are low (*M. margotmarshae*: n=6, *M. sambiranensis*: n=5, and *M. mamiratra*: n=7) (Extended Data Fig. 4h, Supplementary Table 16). Interestingly, however, and in contrast to all other studied *Microcebus* species, the three candidates seem to lack reproductive seasonality, as oestrous females were already found in late June (*M. mamiratra*) or early August (*M. margotmarshae*), pregnant females were still observed in June (*M. mamiratra*) or even early August (*M. margotmarshae*), and lactating females were still found in June (*M. sambiranensis*) or July (*M. mamiratra*) which coincides with the lean season (= dry season), typically regarded as unfavourable for rearing dependent lemur offspring. In summary, the clear genomic diagnosability and the morphometric differentiation support the current classification of the three candidates as distinct species. However, as our sampling only covers part of the distributions of these candidates (Extended Data Fig. 4a; Supplementary Tables 13 to 16), additional sampling, particularly at distributional margins, will be necessary to rule out that our genetic and/or morphometric data fail to represent an existing cline in character variation.

Based on our sampling, we identify at least two reciprocally monophyletic evolutionarily significant units within *M. mamiratra*, corresponding to the mainland population, which occurs in the Galoko Kalobinono NPA, and the population on Nosy Be, which occurs in the small Lokobe NP (Extended Data Fig. 4b). Both *M. margotmarshae* and *M. sambiranensis* should be considered a single evolutionarily significant unit, presumably occurring in the Manongarivo SR and Sahamalaza-Ile Radama NP, respectively.

M. arnholdi (Louis et al., 2008), *M.* sp. 1 (Sgarlata et al., 2019):

M. arnholdi was described at Montagne d'Ambre NP and SR based on molecular diagnosability in mitochondrial D-loop and PAST sequence fragments of ten individuals compared to previously described *Microcebus* species[3]. It is a microendemic species restricted to these regions and the neighbouring forest fragment Antsoroby. The supposed presence of a putative new species (*M.* sp. 1) about 140 km south-east of M. d'Ambre,

between the rivers Bemarivo and Manambato, has been suggested by Sgarlata et al.[21] (Extended Data Fig. 5a). The gap between the two distributions appears to be real and not due to sampling since the majority of forest fragments has been sampled across northern Madagascar. Species delimitation tests performed in Sgarlata et al. between *M. arnholdi* and *M.* sp. 1 were based on two mitochondrial loci. Here, we show that the original delimitation defined in Sgarlata et al. is not supported by nuclear genomic data. Instead, phylogenetic inference identified major genomic differentiation between northern and southern *M. arnholdi* populations (Extended Data Fig. 5b), which is why we decided to carry out species delimitation tests by classifying the northern populations as *M. arnholdi* and the southern populations as *M.* sp. 1. Note, therefore, that the geographic definition of *M.* sp. 1 used herein is different from the one used in Sgarlata et al.

Patterns of genomic IBD between the candidate pair appear to be an extension of within-candidate patterns, in line with an intraspecific model of diversification (Extended Data Fig. 5d; Supplementary Table 2). While our analyses indicate that the two candidates are reciprocally monophyletic (Extended Data Fig. 5b), they do not form distinct genomic clusters, showing admixed ancestry for individuals sampled in Binara Forest (Extended Data Fig. 5c). The genealogical divergence index is inconclusive to delimit this candidate pair, although relatively small (Extended Data Fig. 5e, Supplementary Table 3). Hypervolume overlap in morphometry is high, and patterns of morphometric IBD are continuous, supporting an intraspecific model as well (Extended Data Fig. 5f, Supplementary Tables 4 and 5). Climatic niche overlap (Schoener's D) is comparably low and does not deviate significantly from a null distribution (Extended Data Fig. 5g; Supplementary Table 6), which can be explained by the relatively large spatial distribution of the two candidates at different elevations. Data on reproductive activity are too limited to draw conclusions (average monthly sample size of three for *M. arnholdi* and eight for *M.* sp. 1; Extended Data Fig. 5h; Supplementary Table 16). In summary, patterns of IBD in genomic and morphometric data, which are supported by admixed ancestry, indicate that *M.* sp. 1 does not represent a distinct species but should be synonymised under *M. arnholdi*. Given that our sampling covers the two candidates' known distributions and their margins extensively (Extended Data Fig. 5a; Supplementary Tables 13 to 16), it is unlikely that additional sampling will challenge this conclusion.

Based on our sampling, we identify at least four reciprocally monophyletic evolutionarily significant units in this group, corresponding to the Binara population (within the Loky-Manambato Protected Harmonious Landscape), the Montagne d'Ambre and Antsoroby populations (the former within the Montagne d'Ambre NP), the southern Analalava/Bezavona/Salafaina/Ambohitandrina populations (occurring in non-protected areas) and the central populations inhabiting part of the Corridor of Marojejy-Anjanaharibe Sud-Tsaratanana Nord (COMATSA Nord PA) (Extended Data Fig. 5b).

M. boraha (Hotaling et al., 2016)*, M. simmonsi* (Louis et al., 2006):

M. simmonsi was described based on molecular diagnosability in the mitochondrial D-loop region of nine individuals at Betampona SR and Zahamena NP[2]. It occurs in lowland humid forests of Madagascar's east coast between the Anove River in the north and the Ivondro River in the south. *M. boraha* is confined to its type locality on Île Ste. Marie (Nosy Boraha; Extended Data Fig. 6a) and was described due to monophyly (inferred from two mitochondrial loci), distinct clustering (inferred from four nuclear loci) and multispecies coalescent (MSC)-based species delimitation analyses (using both mitochondrial and nuclear loci)[22]. Notably, the MSC is known to confound population structure with speciation[23,24], and these analyses only included *M. simmonsi* individuals from Tampolo but not from the northern parts of its distribution.

Patterns of IBD in genomic data are inconclusive for delimitation of the candidate pair, as neither the intra- nor the interspecific model are clearly rejected (Extended Data Fig. 6d; Supplementary Table 2). However, genetic distances between individuals of the two candidates are lower than those found among *M. simmonsi* individuals alone when considering similar geographic distances (Extended Data Fig. 6d). Furthermore, our analyses indicate that the two candidates are not reciprocally monophyletic, as *M. boraha* and a *M. simmonsi* lineage north of the Simianona River form a clade that is sister to the remaining *M. simmonsi* (Extended Data Fig. 6b). This is also supported by the clustering analysis (Extended Data Fig. 6c). The *gdi* is intermediate for this candidate pair (Extended Data Fig. 6e, Supplementary Table 3). Because comprehensive morphometric, climatic and reproductive activity data are lacking for *M. boraha* (Supplementary Fig. 22bcd; Supplementary Tables 13 to 16), these lines of evidence can not be integrated. In summary, however, genomic analyses provide sufficient evidence to synonymise *M. boraha* under *M. simmonsi*. Notably, we naively labelled individuals at Ambodiriana as *M. simmonsi*, following Poelstra et al.[16]. If these are labelled as *M. boraha* instead (as indicated by phylogenetic inference), we no longer observe a lack of reciprocal monophyly and mixed clusters (Extended Data Fig. 6bc). However, we still find a relatively continuous IBD pattern (indicated by the point cloud in Extended Data Fig. 6d, bottom), and there is only a comparably small number of substitutions separating the two candidates in the phylogeny (Extended Data Fig. 6b). Given that the genomic differentiation, although detectable, is low and not supported by differentiation in any other trait (albeit this is due to lack of data) and we lack sampling between Ambodiriana and southern *M. simmonsi* populations to test for clinal variation (Extended Data Fig. 6a), we suggest synonymising *M. boraha* in this case as well until more evidence becomes available. We come to this conclusion despite the limited data because we aim to consistently delimit species across the entire genus *Microcebus*, using a conservative approach in the sense that we do not reject a single-species

null hypothesis until there is convincing evidence for differentiation (i.e., genomic data and additional lines of evidence). In any case, the sampling gap between the rivers Simianona and Maningory (Extended Data Fig. 6a) needs to be addressed to ultimately clarify the taxonomic relationship of the two candidates.

Based on our sampling, we identify at least three genetically differentiated, reciprocally monophyletic evolutionarily significant units in this group, i.e., a northern *M. simmonsi* lineage between the rivers Anove and Simianona, a southern *M. simmonsi* lineage between the rivers Ivondro and Maningory and the population on Île St. Marie (corresponding to *M. boraha*) (Extended Data Fig. 6b). Only one of these units (i.e., the southern *M. simmonsi* lineage) occurs in protected areas, i.e., Zahamena NP and Betampona Special Nature Reserve (SNR). The distributions of the other two are not protected to date.

M. jollyae (Louis et al., 2006), *M. marohita* (Rasoloarison et al., 2013), *M. gerpi* (Radespiel et al., 2012):

M. jollyae was described based on molecular diagnosability in the mitochondrial D-loop region of three individuals at Kianjavato and Mananjary[2]. Similarly, the description of *M. gerpi* is based on molecular diagnosability in two mitochondrial loci (COII and D-loop) of 14 individuals at Sahafina Forest compared to previously described *Microcebus* species (including *M. jollyae*)[25]. Notably, the authors also found that *M. gerpi* individuals (n = 7) had significantly longer tails than *M. jollyae*. Finally, *M. marohita* was described from three individuals at Marohita Forest (District de Marolambo) due to monophyly (inferred from two mitochondrial and four nuclear loci) and distinct clustering (inferred from four nuclear loci)[4,26]. These analyses included *M. jollyae* but not *M. gerpi*.

The three candidates are all microendemics of the lowland humid forests of Madagascar's east coast, with disjunct distributions that are separated by large rivers. The distribution and population genomics of *M. gerpi* have recently been explored in detail[27,28]. It occurs between the Ivondro River in the north and the Mangoro River in the south, while being restricted by an elevational limit at around 600 m above sea level. In contrast, *M. marohita* and *M. jollyae* are only poorly sampled, with potential distributional limits presented by the rivers Mangoro and Manapatrana/Mananara, respectively. Accordingly, our sampling covers the entire distribution of *M. gerpi* but only includes two and one samples of *M. jollyae* and *M. marohita*, respectively (Extended Data Fig. 7a; Supplementary Tables 13 to 16).

Patterns of IBD in genomic data clearly support an interspecific model when comparing *M. gerpi* and *M. jollyae* (Extended Data Fig. 7d, Supplementary Table 2). For *M. marohita,* the statistical test could not be conducted due to limited sampling (Supplementary Table 13), but

pairwise genetic distance between *M. jollyae* and *M. marohita* individuals are lower than those observed among *M. gerpi* individuals alone (Extended Data Fig. 7d). In line with van Elst et al.[28], our analyses further indicate that the three candidates are reciprocally monophyletic (Extended Data Fig. 7b) and form distinct genomic clusters (Extended Data Fig. 7c). Notably, population structure within *M. gerpi* is prioritised in admixture analysis when assuming three clusters (K = 3; Extended Data Fig. 7c). Moreover, *gdi* values between all species pairs are particularly high (Extended Data Fig. 7e, Supplementary Table 3). Hypervolume overlap in morphometry and overlap in climatic niches (Schoener's D) could not be quantified with respect to *M. marohita* due to limited sampling (Supplementary Tables 14 and 15). However, we observe low overlap in morphometry between *M. gerpi* and *M. jollyae* (Extended Data Fig. 7f; Supplementary Table 4). Corresponding patterns of IBD are inconclusive for species delimitation (Supplementary Table 5). Conversely, overlap in climatic niches is high and does not differ significantly from a null distribution (Extended Data Fig. 7g, Supplementary Table 6), which can be explained by the proximity of distributions that appear to be separated only by riverine barriers. Data on reproductive activity are limited (average monthly sample size of 3 for both *M. gerpi* and *M. jollyae*, respectively; Supplementary Table 16) but indicate an overlap between the two species as well (Extended Data Fig. 7h). Patterns of IBD, genomic diagnosability and morphometric differentiation support the classification of *M. gerpi* as a separate species from *M. jollyae*. Conversely, we advocate synonymising *M. marohita* under *M. jollyae* until more comprehensive sampling, which is urgently required, becomes available given that (1) both species were only described from a single locality each, (2) admixture and genomic IBD analyses suggest lower differentiation between *M. marohita* and *M. jollyae* than within *M. gerpi*, and (3) we currently have no evidence for differentiation in any other trait, albeit this is due to lack of data. We come to this conclusion despite the limited data because we aim to consistently delimit species across the entire genus *Microcebus*, using a conservative approach in the sense that we do not reject a single-species null hypothesis until there is convincing evidence for differentiation (i.e., genomic data and additional lines of evidence). Our conclusion to treat *M. gerpi* as a separate species most likely also holds when comparing it to a candidate comprised of *M. jollyae* and *M. marohita* individuals of this study (i.e., after synonymising them) given that genetic distances plotted against geographic distances between individuals of *M. gerpi* and *M. jollyae* or *M. marohita* form a single point cloud (Extended Data Fig. 7d, bottom) and that data for *M. marohita* are very limited anyway. It is worthy of note that the genomic analysis of van Elst et al. found particularly high genomic differentiation between *M. gerpi* populations north and south of the Rianila River. However, preliminary investigations (Schüßler, Rakotondravony, Radespiel, unpubl. data) did not find any significant differentiation in morphometry or climatic niches between these two lineages, which is why we do not consider them distinct species.

The detailed population genomic analysis in van Elst et al. suggests that *M. gerpi* is comprised of at least four differentiated, reciprocally monophyletic evolutionarily significant units, namely a northern lineage at Sahamamy/Anjahamana//Andobo, a central lineage at Vohiposa/Sahafina and two southern lineages at Ambodisakoana and Antanambao, respectively (Extended Data Fig. 7b). Due to the limited sampling, *M. jollyae* and *M. marohita* have to be each considered a single evolutionarily significant unit until more comprehensive studies are available. At present, *M. gerpi* occurs in the Sahafina NPA but none of the other units occur in formally protected areas.

M. macarthurii (Radespiel et al., 2008), *M. jonahi* (Schüßler et al., 2020):

M. macarthurii was described from three individuals at Anjiahely based on molecular diagnosability in three mitochondrial loci compared to previously described *Microcebus* species[29]. The description of *M. jonahi* was based on morphometric and genomic species delimitation analyses performed specifically in comparison with *M. macarthurii* and using extensive sampling of both species[16,17].

The two candidates occur allopatrically in the humid forests of northeastern Madagascar (Extended Data Fig. 8a). Their distributions are likely separated by the Rantabe River. While *M. macarthurii* occurs north of it up to the Antainambalana River, *M. jonahi* is distributed along its southern shore down to the Anove River. Notably, however, the species of *Microcebus* individuals occurring between the rivers Rantabe and Voloina has not been identified by molecular data so far.

Patterns of IBD in genomic data are inconclusive for delimitation as neither the intra- nor the interspecific model are clearly rejected, but genetic distances between candidates appear to be higher than those within, when considering similar geographic distances (Extended Data Fig. 8d; Supplementary Table 2). Furthermore, our results confirm that the two candidates are reciprocally monophyletic (Extended Data Fig. 8b), form distinct genomic clusters (Extended Data Fig. 8c) and have intermediate to high *gdi* (Extended Data Fig. 8e, Supplementary Table 3). As already indicated by Schüßler et al.[17], hypervolume overlap in morphometry is comparably low (no significant pattern of IBD is detected; Extended Data Fig. 8f; Supplementary Tables 4 and 5). Overlap in climatic niches (Schoener's D) is intermediate and deviates significantly from a null distribution (Extended Data Fig. 8g; Supplementary Table 6), which can be explained by the proximity of species distributions, which are separated only by a single river. The reproductive schedules of both species seem to be synchronous and there is no evidence for differentiation (Extended Data Fig. 8h; see also Schüßler et al.), but the underlying average monthly sample sizes are low (*M. jonahi*: n=12; *M. macarthurii*: n=5;

Supplementary Table 16). The genomic diagnosability and morphometric differentiation support the current classification of *M. macarthurii* and *M. jonahi* as distinct species. However, further data are needed to ultimately exclude the possibility that the two candidates represent lineages on opposite ends of a genetic cline since *M. macarthurii* has only been sampled from a single locality to date (Extended Data Fig. 8a).

Based on our sampling, genomic analyses do not indicate the presence of differentiated lineages and therefore separate evolutionarily significant units within the two species, which is consistent with Poelstra et al.[16] (Extended Data Fig. 8b). We therefore suggest treating each species as a single unit until more data become available. Both species currently occur in protected areas (*M. macarthurii* in Makira Natural Park; *M. jonahi* in Makira Natural Park and Mananara Nord NP).

M. manitatra (Hotaling et al., 2016), *M. ganzhorni* (Hotaling et al., 2016), *M. murinus* (Miller, 1777):

M. murinus is the earliest recognized *Microcebus* species[30]. *M. ganzhorni* and *M. manitatra* were described from previously considered *M. murinus* populations at Mandena and Bemanasy Forest, respectively, due to monophyly (inferred from two mitochondrial loci), distinct clustering (inferred from four nuclear loci) and MSC-based species delimitation analyses (using both mitochondrial and nuclear loci)[22]. Notably, the MSC is known to confound population structure with speciation[23,24].

M. murinus is the most widely distributed species in the entire genus, occurring in dry, gallery and, to some extent, spiny forests along Madagascar's west coast between the Sofia River in the northwest and the Mandena region around Fort Dauphin in the south. Due to the wide distribution of *M. murinus*, we split it into three candidates corresponding to samples north of the Manambaho River (northern lineage), between the rivers Tsiribihina and Onilahy (central lineage) and east of the Mandrare River (southern lineage; Extended Data Fig. 9a). However, genomic data for the southern lineage are restricted to few low-quality samples (Supplementary Tables 13 and 18), which is why most delimitation analyses were only conducted for the northern and central lineages. Notably, these lineages are separated by a wide sampling gap (Extended Data Fig. 9a; Supplementary Table 15) with no confirmed sightings of *M. murinus*[13], which may indicate an actual distributional gap. *M. manitatra* and *M. ganzhorni* are both microendemics in the southern part of the range of *M. murinus* (Extended Data Fig. 9a).

When treating *M. murinus* (north) and *M. murinus* (central) as separate candidates, patterns of IBD in genomic data are mostly inconclusive for delimitation (Extended Data Fig.

9d; Supplementary Table 2). Notably, however, treating *M. murinus* (north) and *M. murinus* (central) together as a single candidate supports a synonymisation of *M. manitatra* and *M. ganzhorni* under *M. murinus* (Supplementary Table 2). Furthermore, our analyses indicate that none of the four candidates are reciprocally monophyletic (Extended Data Fig. 9b), as *M. ganzhorni* is nested within *M. manitatra,* and both are nested within *M. murinus* (central). Together, the three candidates form the sister clade to *M. murinus* (north). While assuming two clusters (K = 2) suggests admixed ancestry between *M. murinus* (central) and *M. murinus* (north), additional signatures of gene flow are detected between *M. murinus* (central), *M. murinus* (south) and *M. manitatra* as well as between *M. ganzhorni* and *M. manitatra*, when setting the number of clusters to three (K = 3) and four (K = 4), respectively (Extended Data Fig. 9c). In accordance with these findings, the *gdi* is particularly low between *M. manitatra* and *M. ganzhorni* (Extended Data Fig. 9e, Supplementary Table 3). Interestingly, however, in other comparisons, *gdi* calculated from θ of *M. murinus* (north), *M. manitatra* and *M. ganzhorni* are comparably high (Supplementary Table 3), which can result from inbreeding reducing the effective population size (and θ), thus biasing the *gdi* upwards. While hypervolume overlap in morphometry is comparably high between *M. murinus* lineages (Extended Data Fig. 9f, Supplementary Table 4), overlap in climatic niches (Schoener's D) is low to intermediate and differs significantly from a null distribution (as can be expected given the wide distribution of *M. murinus*; Extended Data Fig. 9g, Supplementary Table 6). Estimates with respect to *M. manitatra* and *M. ganzhorni* are not available due to the very limited data (Supplementary Tables 14 and 15). Reproductive data are available for *M. murinus* (central) and *M. murinus* (north), indicating that females enter oestrus about 1 – 2 months earlier in the northern than in the central clade (Extended Data Fig. 9h). This discrepancy can likely be explained by differences in day length dynamics and seasonal climatic changes[19]. For *M. manitatra* and *M. ganzhorni*, reproductive data are mostly lacking (Supplementary Table S16). Finally, advertisement calls of the three candidates *M. murinus* (north), *M. murinus* (central) and *M. ganzhorni* show similar contours of the fundamental frequency in comparison to other *Microcebus* species (Supplementary Fig. 7)[31,32]. Whereas all advertisement calls of these candidates are characterised by an initial modulation followed by several up and down modulated syllables, calls of *M. ravelobensis*[31], *M. lehilahytsara*[31], *M. mamiratra*[32] and *M. margotmarshae* (unpublished data), for instance, consist only of one to three modulated syllables. Hypervolume overlap in acoustic profiles was lowest between *M. murinus* (north) and *M. ganzhorni* (Supplementary Table 25). *M. murinus* (central), however, showed similar levels of overlap to *M. murinus* (north) and *M. ganzhorni,* suggesting a gradient from north to south which is consistent with a single-species hypothesis for this candidate group. In summary, patterns of IBD supported by the phylogenetic nestedness of *M. manitatra* and *M.*

ganzhorni within *M. murinus* and the apparent gene flow strongly suggest synonymising the two candidates under *M. murinus*.

It is worthy of note that the deepest split in the phylogeny of this clade can be found between northern *M. murinus* and the central lineage plus *M. ganzhorni* and *M. manitatra,* raising the question whether these deserve separate species status. Although our IBD based test is inconclusive to delimit these lineages, the plot of geographic against genetic distances reveals a relatively continuous pattern of IBD when considering comparisons within *M. murinus* (central), within *M. murinus* (north) and between the central and northern lineage (Extended Data Fig. 9d), indicating that genetic distances can be explained by geographic distribution rather than speciation. This is also supported by admixed ancestry of Bombetoka individuals at $K = 2$ (Extended Data Fig. 9c), small branch lengths separating the central from the northern lineage (compared to the number of substitutions present within the central/southern clade; Extended Data Fig. 9b), and the potential sampling gap between the distributions of the two lineages. Finally, overlap in morphometry and acoustic profiles is high, and the observed differences in climatic space and reproductive activity mentioned above can likely be explained by the large distribution of this species, covering almost the entire north-south axis of Madagascar (Extended Data Fig. 9b). Comparing individuals from the northern end and the more southern part of this distribution inevitably results in the detection of differences in climatic space and potentially reproductive activity (which can be affected by climate). Accordingly, our findings do not support a classification as distinct species. Additional sampling will definitely help further characterising genetic structure and variation in traits such as morphometry, reproductive activity or acoustic communication within *M. murinus*. However, it is unlikely to challenge our conclusion regarding its taxonomy given the clear evidence for gene flow across its range and because our geographically informed approaches alleviate the effect of uneven and/or sparse sampling.

Based on our sampling, genomic analyses identify at least four differentiated, reciprocally monophyletic evolutionarily significant units in this group, corresponding to a northern *M. murinus* lineage between the rivers Sofia and Betsiboka, a northern lineage south of the Betsiboka stretching towards the Tsingy de Namoroka, the central lineage around Menabe-Antimena NP and the southern lineage around Fort Dauphin which includes the candidates *M. manitatra* and *M. ganzhorni* (Extended Data Fig. 9b). While the former three units all occur in protected areas (Ankarafantsika NP, Tsingy de Namoroka NP and Menabe-Antimena NP, respectively), the southern lineage is only protected in the small Mandena Conservation Zone.

M. ravelobensis (Zimmermann et al., 1998), *M. bongolavensis* (Olivieri et al., 2007), *M. danfossi* (Olivieri et al., 2007):

M. ravelobensis was described from Ampijoroa in central eastern Madagascar based on morphometric differentiation of 27 individuals to sympatric *M. murinus*[33]. The descriptions of *M. bongolavensis* and *M. danfossi* were based on minor differentiation in morphometry as well as molecular diagnosability in two mitochondrial loci (COII and cytochrome b) of three and seven individuals at Ambodimahabibo and Ambarijeby (Province of Mahajanga), respectively, compared to the other two species in this group[34].

The three candidates are distributed in the dry deciduous forests of northwestern Madagascar, separated by the two large rivers Mahajamba (*M. ravelobensis* - *M. bongolavensis*) and Sofia (*M. bongolavensis* - *M. danfossi*) (Extended Data Fig. 10a).

We find a clear rejection of a single-species model in analyses of genomic IBD when comparing *M. danfossi* with the other two candidates but inconclusive results for the comparison of *M. bongolavensis* and *M. ravelobensis* (Extended Data Fig. 10d, Supplementary Table 2). Moreover, the three candidates are reciprocally monophyletic (with comparably short branch lengths between *M. ravelobensis* and *M. bongolavensis* though; Extended Data Fig. 10b) and form distinct clusters (Extended Data Fig. 10c). While the *gdi* is high when considering *M. danfossi*, it is intermediate between *M. bongolavensis* and *M. ravelobensis* (Extended Data Fig. 10e, Supplementary Table 3). The low but detecSupplementary Table genomic differentiation between the latter two candidates is not supported by other lines of evidence: Hypervolume overlap in morphometry and overlap in climatic niches (Schoener's D) are high or intermediate to high, respectively (Extended Data Fig. 10fg; Supplementary Tables 4 and 6), and morphometric patterns of IBD are continuous and therefore concordant with an intraspecific model (Supplementary Tables 1 and 5). In addition, using extensive sampling we do not detect any differentiation in reproductive schedules but a synchronous activation of reproductive activity of *M. bongolavensis* and *M. ravelobensis* (Extended Data Fig. 10h). More specifically, enlarged testes can be observed in both species starting around July, and although our sampling does not cover months prior to this for *M. bongolavensis* (in contrast to *M. ravelobensis*), the absence of regressed testes in both species starting approximately in September further supports the hypothesis that reproductive activity in males begins around the same time. Similarly, the appearance of female oestrus and pregnancy seems to be synchronous for the two candidates. In contrast, *M. danfossi* appears to start reproductive activity several months earlier (i.e., earlier pregnancies and testes growth; Extended Data Fig. 10h; see also Rina Evasoa et al.[19]). Finally, hypervolumes of acoustic profiles of alert calls are largely overlapping as well (Supplementary Fig. 7d, Supplementary Table 25), which is further supported by the

sonograms showing a more similar contour of the fundamental frequency between *M. ravelobensis* and *M. bongolavensis* in comparison to *M. danfossi* (Supplementary Fig. 7c; see also Hasiniaina et al.[32]). In summary, these results suggest synonymising *M. bongolavensis* under *M. ravelobensis* as the genomic differentiation, although detecSupplementary Table, is not supported by other taxonomic characters, while confirming the classification of *M. danfossi* as a distinct species due to a rejection of an intraspecific pattern of genomic IBD. This conclusion most likely also holds when comparing *M. danfossi* to a candidate comprising both *M. bongolavensis* and *M. ravelobensis* individuals of this study (i.e., after synonymising them) given that the NRMSE patterns obtained when comparing *M. danfossi* separately to the two other candidates are largely congruent (Extended Data Fig. 10d, top), with a single shared point cloud in the plot of geographic and genetic distances (Extended Data Fig. 10d, bottom). Because our sampling largely covers the known distributions of the candidates including their margins (Extended Data Fig. 10a; Supplementary Tables 13 to 16), it is unlikely that additional sampling will challenge our conclusion regarding their taxonomy.

Notably, delimiting *M. bongolavensis* and *M. ravelobensis* is a particularly difficult case as it mirrors the dispute around different species concepts. That is, the two candidates appear to be genomically diagnosable, but genomic differentiation is low and not supported by other taxonomic characters. They likely represent diverging lineages at an intermediate point along the speciation continuum (i.e., "in the process of speciation"[35]). Here, we argue to synonymise them due to the lack of differentiation in additional taxonomic characters, and because we aim to consistently delimit species across the entire genus *Microcebus*.

We propose to treat each candidate as a separate evolutionarily significant unit (Extended Data Fig. 10b). While *M. ravelobensis* occurs in Ankarafantsika NP (sympatrically with *M. murinus*), *M. bongolavensis* can only be found in the Bongolava Forest Corridor, which does not seem to offer any protection from logging[36]. Similarly, *M. danfossi* occurs in no formally protected area except the Bora SR, which is severely threatened by habitat degradation that likely already led to the local extirpation of *Propithecus tattersalli*[37].

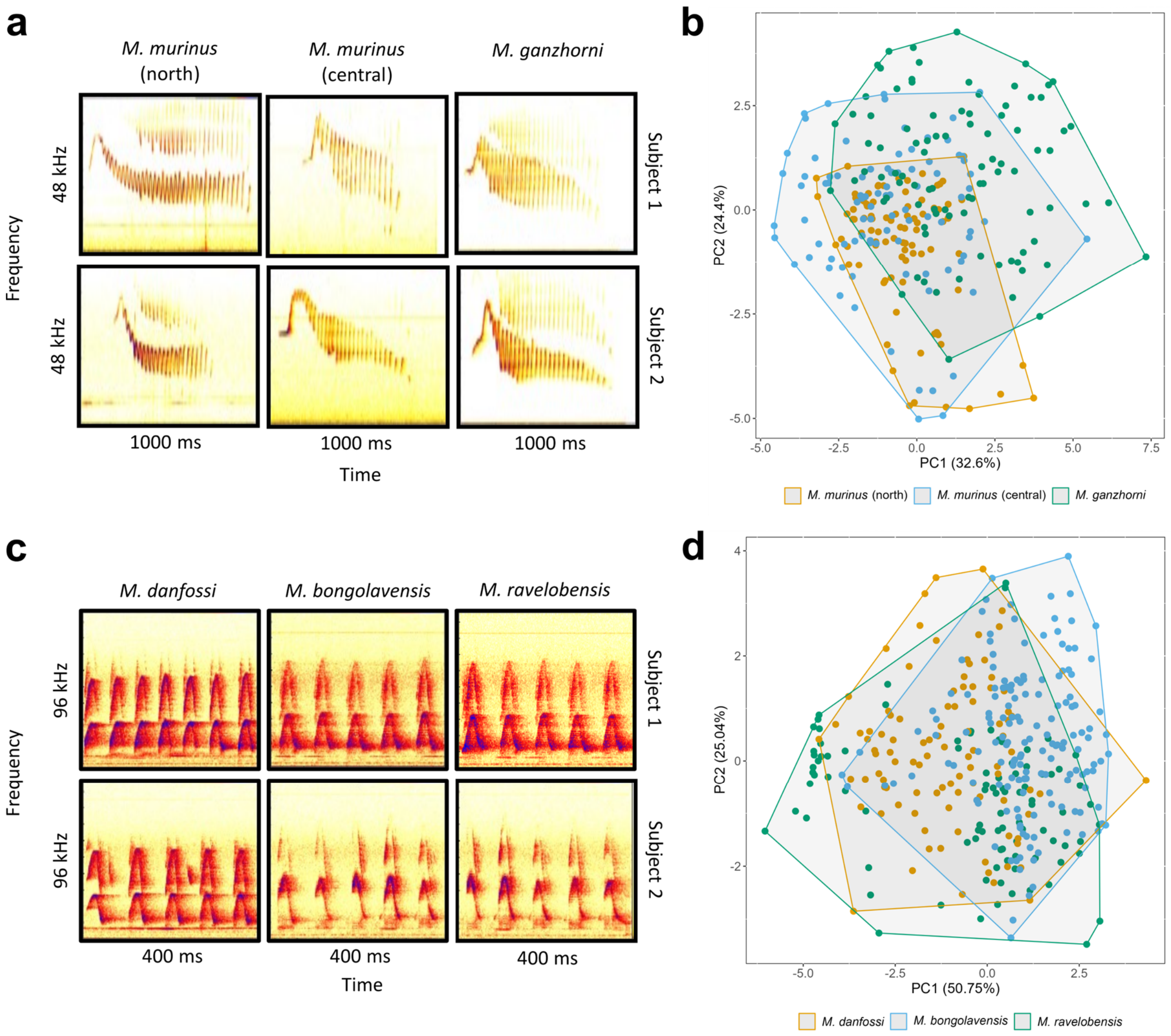

Supplementary Figure 7: Call comparisons in two *Microcebus* candidate groups. Spectrograms of advertisement calls (Trill) of two exemplary individuals (subjects) (**a**) and PCA of *n*-dimensional hypervolumes constructed from acoustic parameters (Supplementary Table S24) (**b**) among the candidates *M. murinus* (north), *M. murinus* (central) and *M. ganzhorni*. Spectrograms of alert calls (Tsak) of two exemplary individuals (subjects) (**c**) and PCA of *n*-dimensional hypervolumes constructed from acoustic parameters (Supplementary Table 24) (**d**) among the candidates *M. ravelobensis*, *M. bongolavensis* and *M. danfossi*.

Singletons: *M. tanosi* (Rasoloarison et al., 2013), *M. tavaratra (*Rasoloarison et al., 2000), *M. griseorufus* (Kollman, 1910):

The three species *M. griseorufus*, *M. tanosi* and *M. tavaratra* are not part of any previously covered candidate group. *M. griseorufus* was intially described based on morphology as a subspecies of what is now considered *M. murinus*[38]*.* It was raised to a full species due to differentiation in external morphological, cranial and dental measurements (six individuals) as well as mtDNA (two individuals) compared to other *Microcebus* species in western

Madagascar[1,13]. Similarly, *M. tavaratra* was described from Ankarana SR based on differentiation in external morphological, cranial and dental measurements (five individuals) as well as mtDNA (two individuals)[13]. Finally, *M. tanosi* was described from ten individuals at the forests of Manantantely and Ivorona (District de Taolagnaro) due to monophyly (inferred from two mitochondrial and four nuclear loci) and distinct clustering (inferred from four nuclear loci)[4,26].

M. griseorufus occurs in the most arid parts of the island, the spiny thickets of southwestern Madagascar. *M. tanosi* may have a rather high ecological capacity, occurring from the transitional lowland forests of southeastern Madagascar to the highland humid forests of Midongy du Sud NP. *M. tavaratra* is a species of the dry deciduous forests of the northern tip of Madagascar, occurring in the distributional gap of *M. arnholdi*. Sampling maps and climatic niche models are given in Supplementary Fig. 8.

Each of the three species is separated from its sister clades by comparably long branches. The status of *M. tanosi* and *M. tavaratra* as distinct species follows from delimitation decisions within their sister clades (see above). In the case of *M. griseorufus*, the relatively large genetic distance to *M. murinus* and the formation of a contact zone in which no gene flow can be found[39] provides strong evidence for its status as a separate species. Based on our sampling, phylogenetic analysis does not indicate the presence of distinct phylogenetic clusters within *M. griseorufus* or *M. tanosi* that could serve as further candidates for species delimitation. Accordingly, we suggest treating these species as single evolutionarily significant units each. Notably, our genomic data only covers the southern parts of their distributions (Supplementary Fig. 8a, Supplementary Table 13), and additional sampling may reveal further structure in these species. In *M. tavaratra*, a major split is observed between the two samples in the forest fragments of Analafiana (the most southern location within its distribution) and the remaining individuals (Supplementary Figs. 1 to 5). We therefore suggest treating the Analafiana population as a separate evolutionarily significant unit compared to the other sampled individuals (see also Salmona[40]). All species can be found in National Parks or other protected areas (e.g., *M. griseorufus*: Tsimanampetsotsa NP, Beza Mahafaly SR, Berenty PR; *M. tanosi*: Andohahela NP, Midongy du Sud NP; *M. tavaratra*: Ankarana SR, Analamerana SR).

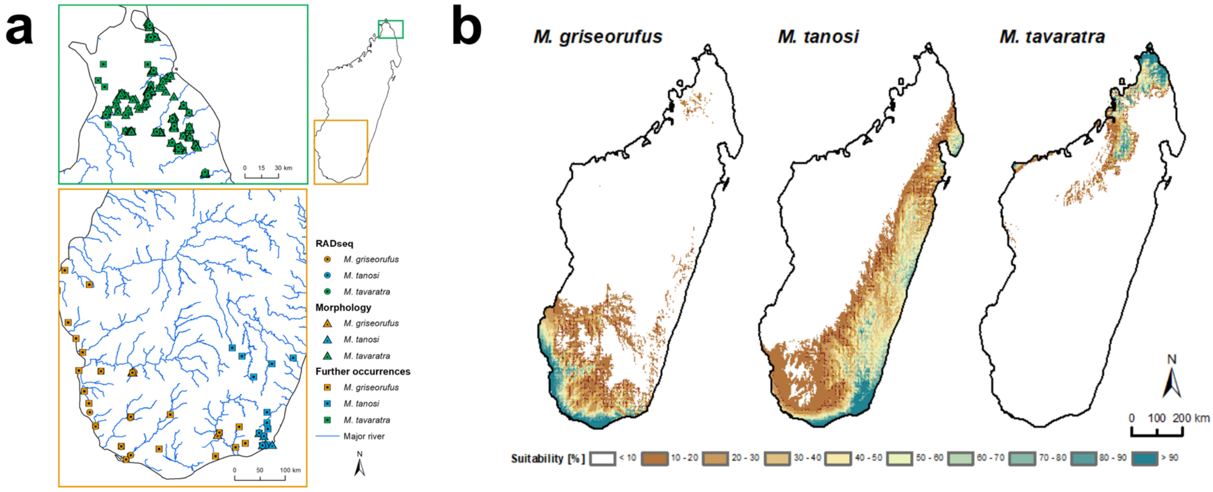

Supplementary Figure 8: Sampling (**a**) and climatic niche models (**b**) of *M. griseorufus*, *M. tanosi* and *M. tavaratra*.

Divergence time estimation (Supplementary Figs. 9 to 14)

We inferred divergence times under a MSC model in BPP, averaging four independent runs of 900,000 generations with a burn-in of 100,000. Convergence of chains was reached for most nodes as indicated by effective sample sizes (ESS) larger than 200 and by median node heights (Supplementary Figs. 9 to 12). Only some of the deeper nodes in the phylogeny with relatively short associated branch lengths (e.g., nodes o, p, h and j) did not reach convergence across all chains with respect to τ and/or θ parameters (Supplementary Figs. 10 to 12).

We converted τ to absolute time using both point estimates of mutation rate and generation time and distributions with point estimates as means (Supplementary Figs. 13 and 14; Supplementary Table 7). While conversion with point estimates resulted in small 95% highest posterior density (HPD) distributions, indicating good convergence of chains, accounting for uncertainty in calibrations led to relatively large 95% HPD distributions. The estimation of population sizes from θ parameters is not the focus of this study and was therefore not conducted.

Our findings suggest that the genus *Microcebus* started diversifying about 1.5 million years (Ma) ago during the mid-Pleistocene (Supplementary Figs. 13 and 14) which is supported by other MSC studies (i.e., < 2 Ma ago)[8,16,18,28] but much younger than fossil-calibration based estimates (i.e., ~8 – 10 Ma ago)[7,9,41,42]. The tendency of the latter to inflate divergence times by not accounting for discordant genealogical histories[43], dating sequence divergence instead of speciation events[44,45] and using external, phylogenetically distant fossil calibrations[46] has been discussed before. Particularly the latter has been common practice to date lemur divergences due to the lack of fossils. Notably, our approach may have

underestimated divergence times by not modelling gene flow after divergence[47–49], but it is unlikely that accounting for this would shift the general timing of diversification outside the Pleistocene.

To conclude, our estimates, accounting for the uncertainties as detailed above, suggest that the diversification of the genus *Microcebus* has taken place during the climatically fluctuating conditions of the Pleistocene (i.e., periodic glaciation events and interglacials). As *Microcebus* species, like most other lemurs, are arboreal primates, they largely depend on closed-canopy forest ecosystems, which have likely been widespread at warmer and wetter periods of time (i.e., interglacials and transition times), but periodically shifted towards more open savannah-like or grassy ecosystems during colder and more arid times (glacial maxima)[50,51]. As a result, lineages were likely forced to follow forested habitats to higher elevations or to humid refugia[52,53], which has recently been empirically exemplified for *M. gerpi*, a lowland humid forest microendemic[28].

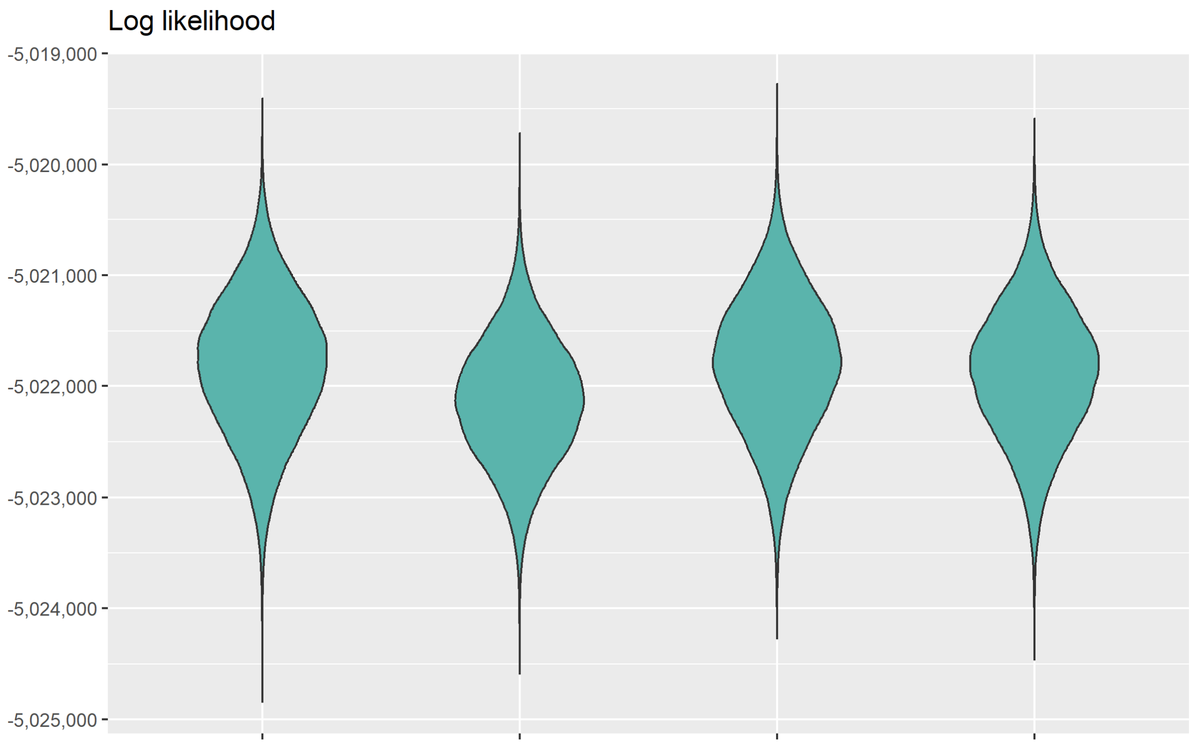

Supplementary Figure 9: Posterior distributions of log likelihood across four BPP chains run for one million generations with a burn-in of 20%.

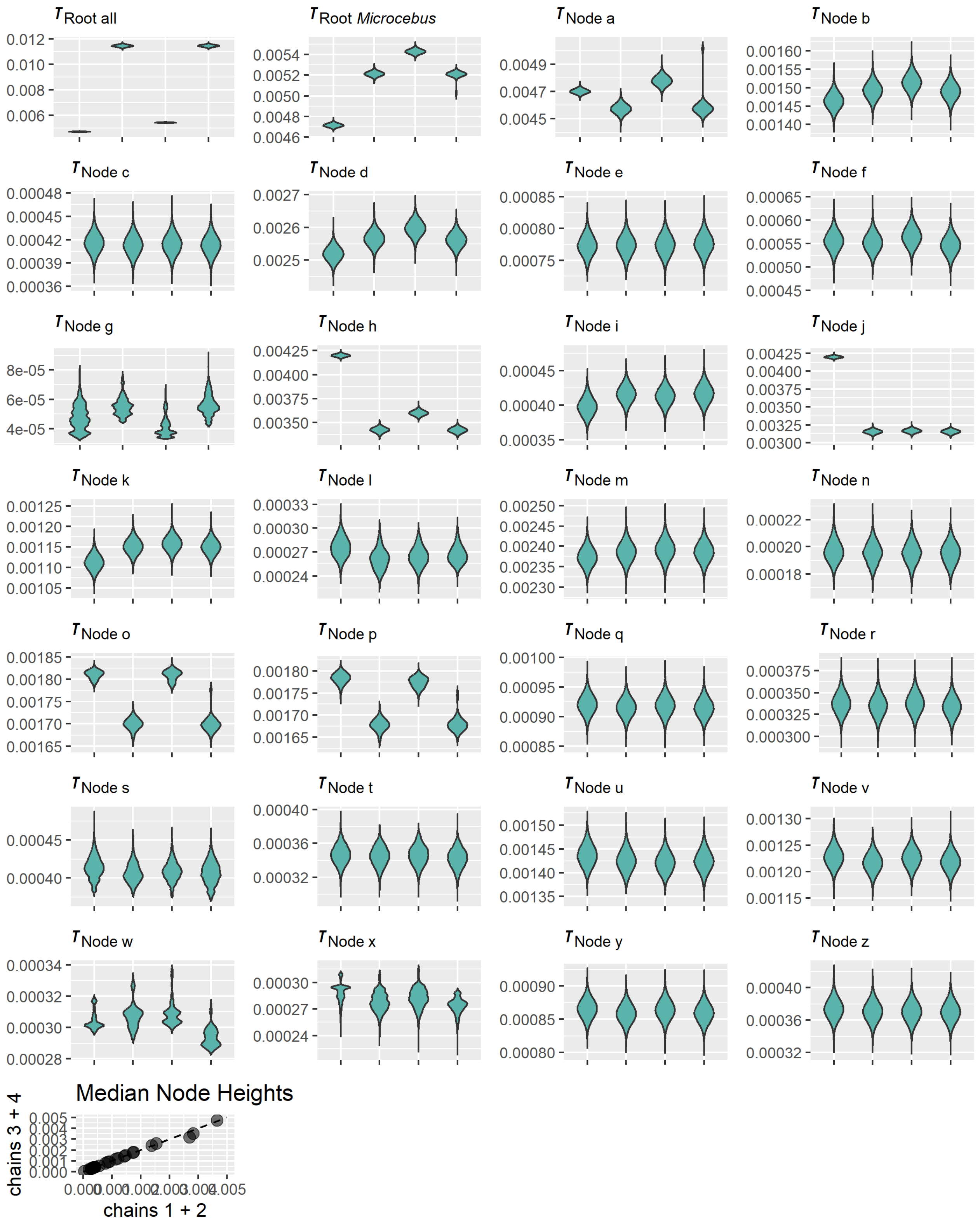

Supplementary Figure 10: Posterior distributions of τ parameters across four BPP chains run for one million generations with a burn-in of 20%. Combined node heights of chains 1 and 2 were compared to those of chains 3 and 4 to check convergence. Node letters correspond to Fig. 2b.

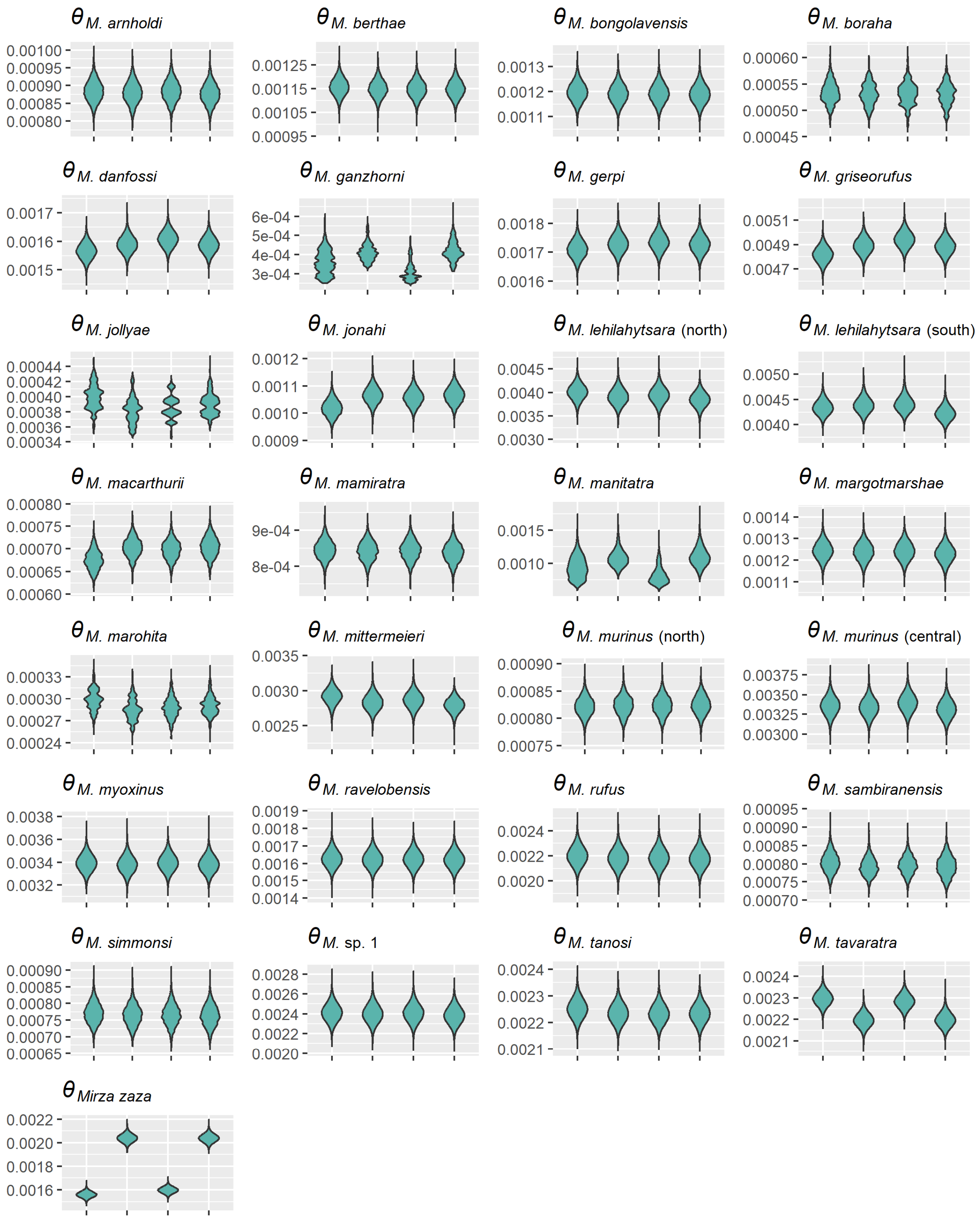

Supplementary Figure 11: Posterior distributions of θ parameters (tips) across four BPP chains run for one million generations with a burn-in of 20%.

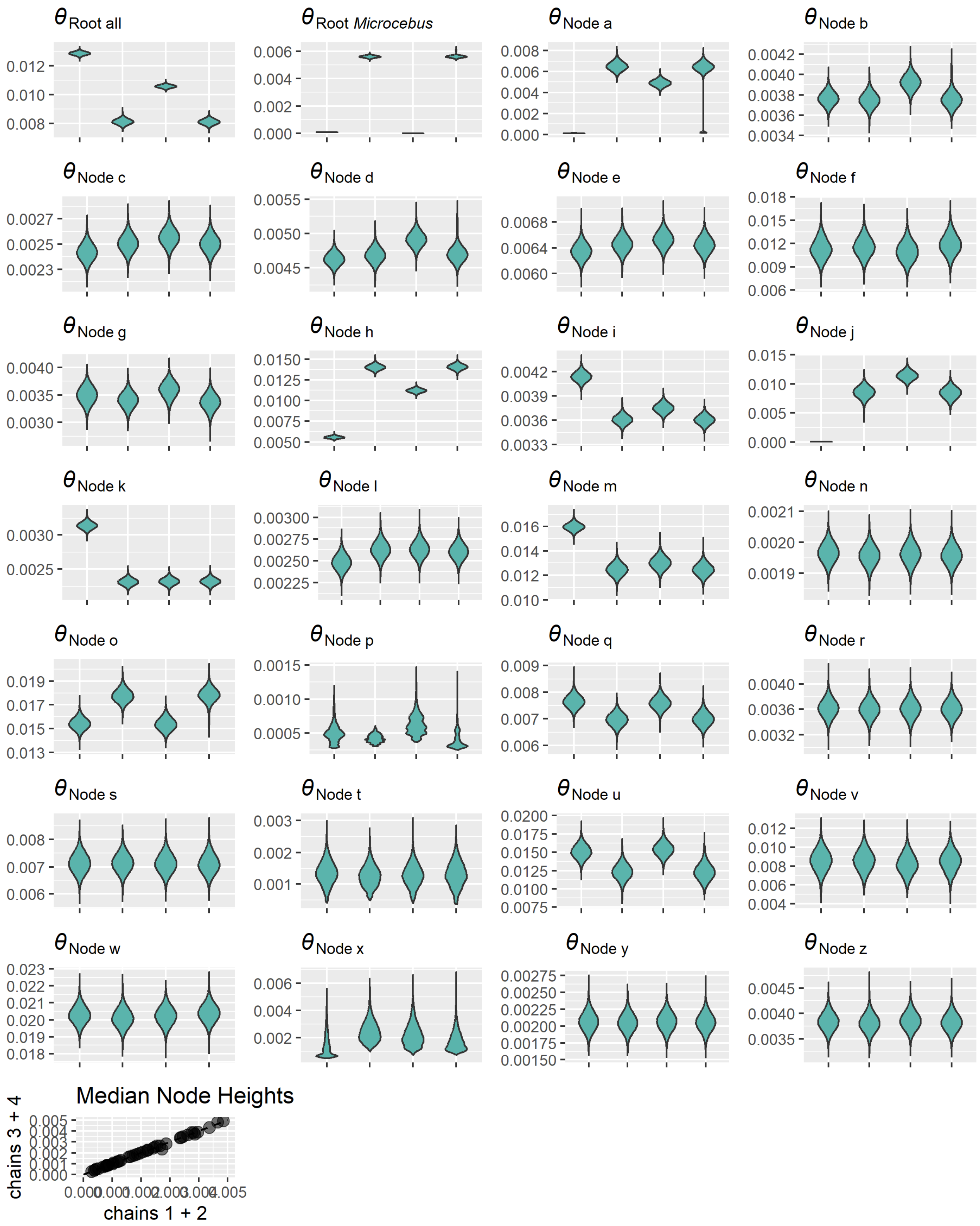

Supplementary Figure 12: Posterior distributions of θ parameters (nodes) across four BPP chains run for one million generations with a burn-in of 20%. Node letters correspond to Fig. 2b. Combined node heights of chains 1 and 2 were compared to those of chains 3 and 4 for all θ parameters (Supplementary Figs. 11 and 12) to check convergence.

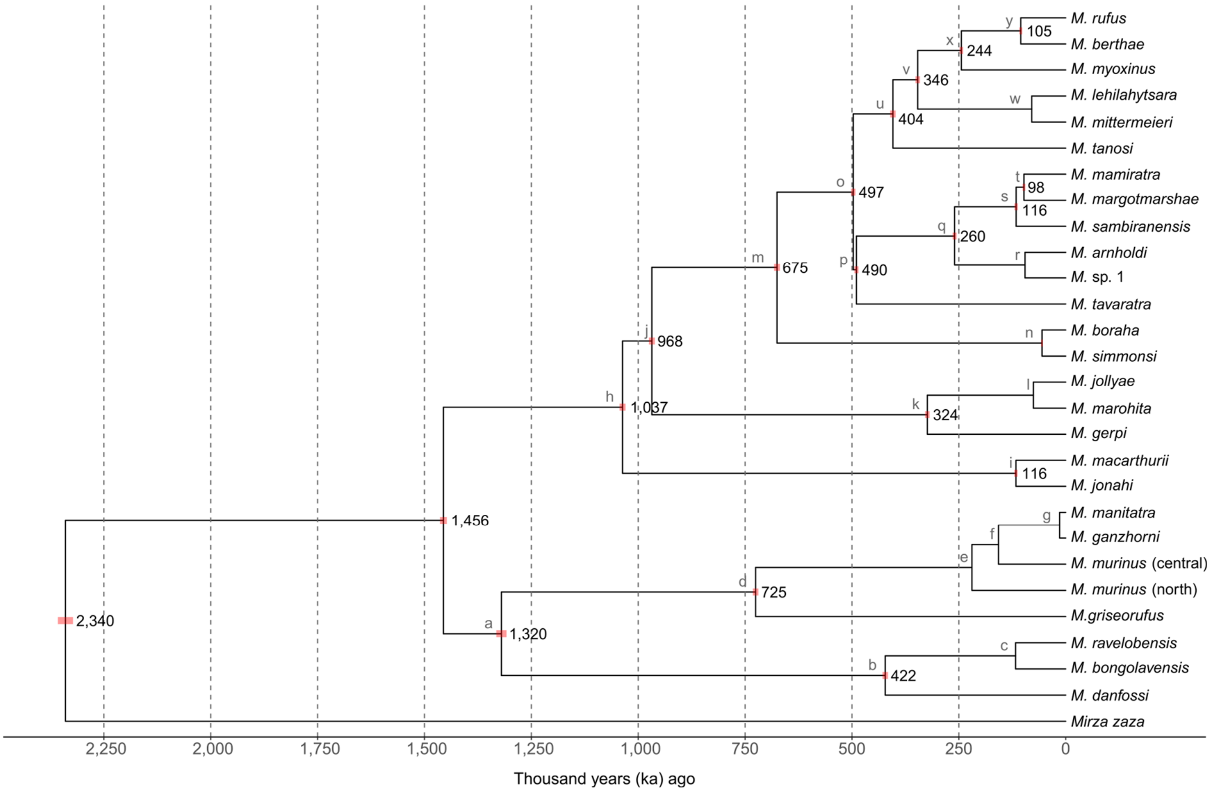

Supplementary Figure 13: Divergence times among *Microcebus* species estimated through a coalescent model in BPP, where the conversion of τ to absolute time was based on a mutation rate of 1.236 x 10^{-8} per site per generation and a generation time of 3.5 years. Red bars indicate 95% highest posterior density distributions. Divergence times among synonymised species are not reported. Nodes are labelled by lower case letters for reference in other analyses.

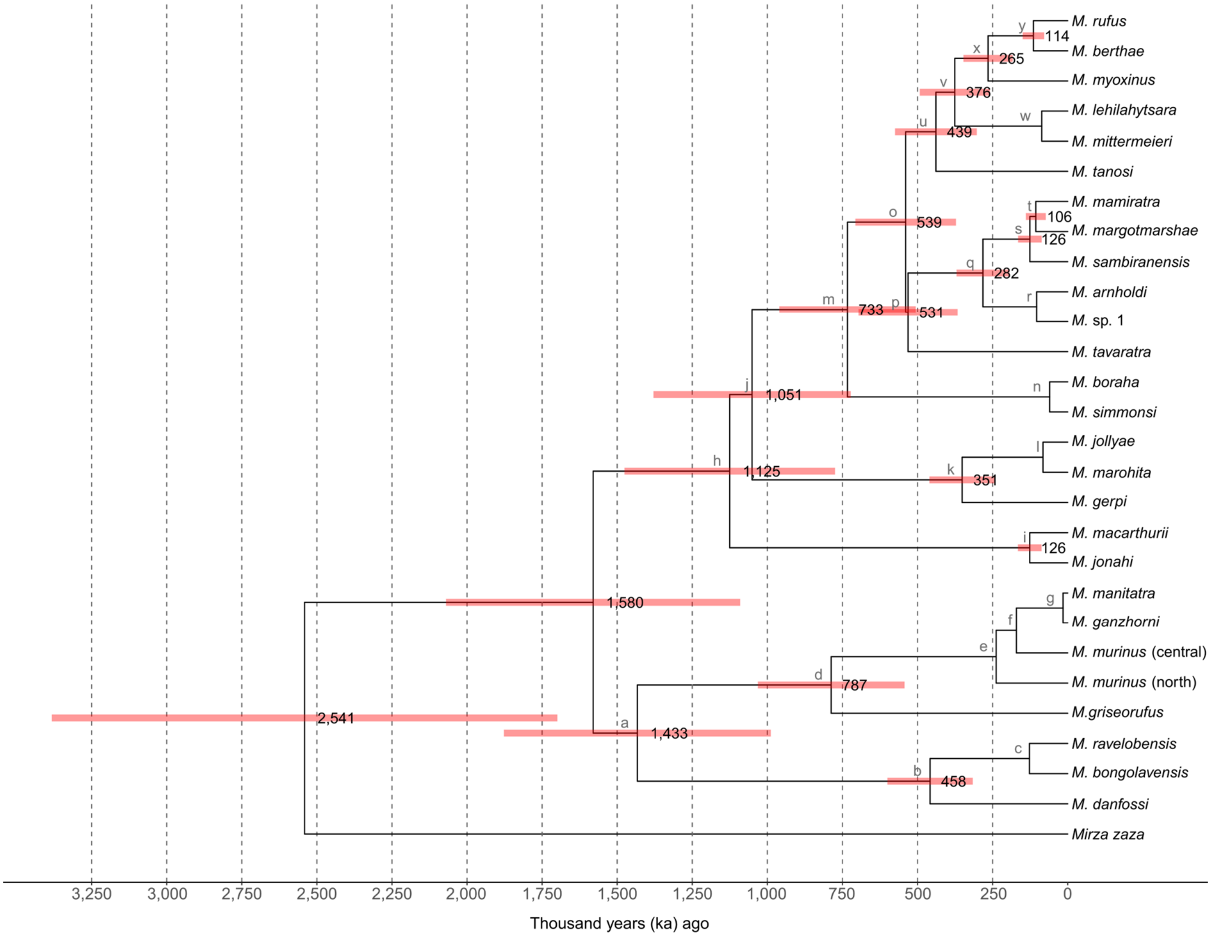

Supplementary Figure 14: Divergence times among *Microcebus* species estimated through a coalescent model in BPP, where the conversion of τ to absolute time was based on a gamma distribution with mean 1.236 x 10^{-8} and variance 0.107 x 10^{-8} and a lognormal distribution with a mean of ln(3.5) and standard deviation of ln(1.16) for mutation rate and generation time, respectively. Red bars indicate 95% highest posterior density distributions. Divergence times among synonymised species are not reported. Nodes are labelled by lower case letters for reference in other analyses.

Biogeographic reconstruction and diversification rate analysis (Supplementary Figs. 15 to 17)

We reconstructed ancestral habitats along the *Microcebus* phylogeny using trait-dependent dispersal models (DEC, BAYAREALIKE, DIVALIKE) with and without jump dispersal (+J) and three different classification schemes (dry vs. humid forest, five major ecoregions as in Yoder et al.[8], and the Köppen-Geiger climate classification[54]). Models with jump dispersal generally performed better than those without (Supplementary Table 8). Following a classification into dry and humid forests, all models suggest that the divergence of these two habitat types (largely analogous to a west vs. east divide, respectively) coincides with the earliest split in the phylogeny (best model: DIVALIKE+J; Supplementary Fig. 15). At least two secondary reversions to dry forests occurred in the humid clade (i.e., *M. berthae*, *M. myoxinus*, *M. tavaratra*). We used the GeoSSE model[55] as implemented in the R package 'diversitree' v0.9-16[56] to examine the effect of habitat type (humid vs. dry) on speciation rates, following Everson et al.[57]. While the best model indicates that humid habitats are associated with higher speciation rates, an equal rates model receives similar support (ΔAIC = 0.19). Accordingly, our data do not provide evidence for a difference in speciation rates associated with habitat type. Testing this hypothesis is likely hampered by the small phylogenetic scale considered here[58]. The most basal split between humid and dry environments is also recovered by the classification into five ecoregions according, while highlighting the dispersal of *M. berthae*, *M. mamiratra*, *M. margotmarshae* and *M. griseorufus* to more specialised ecoregions, i.e., the succulent woodlands, woodland/grassland mosaics and arid spiny bush, respectively (best model: DEC+J; Supplementary Fig. 16). This is also the case for the Köppen-Geiger classification which further differentiated between different tropical, dry, and temperate environments (best model: DEC+J; Supplementary Fig. 17). All three classifications suggest that the ancestral habitat of the genus *Microcebus* spanned both the dry habitats on the west coast and the humid habitats of the east coast of Madagascar, which is doubtful given the large number of microendemics in the genus and the considerably different climatic regimes of these regions. Ultimately, a comprehensive phylogeny that includes other cheirogaleid taxa and their habitat preferences (i.e., *Allocebus trichotis*, *Cheirogaleus* spp., *Mirza* spp., *Phaner* spp.) will be necessary to resolve this question.

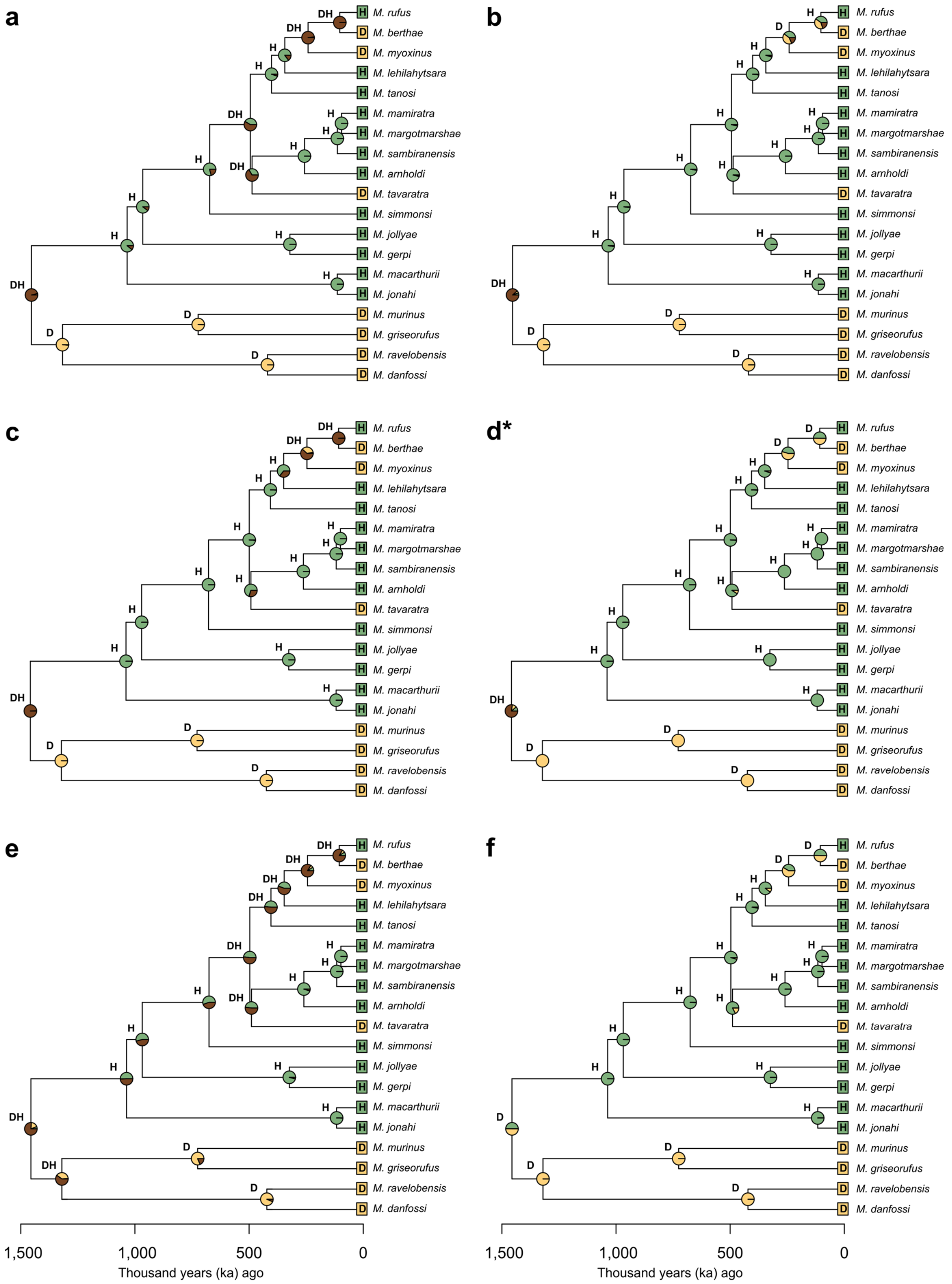

Supplementary Figure 15: Ancestral biogeographic regions of *Microcebus* lineages (D: dry, H: humid), estimated with BioGeoBears under the following models: DEC (**a**), DEC+J (**b**), DIVA-like (**c**), DIVA-like+J (**d**), BAYAREA-like (**e**), BAYAREA-like+J (**f**). Node letters indicate the most likely region. Multiple abbreviations at a single node refer to the combined region (e.g., DH: dry and humid). The best fitting model is indicated by an asterisk. Model details are given in Supplementary Table 8.

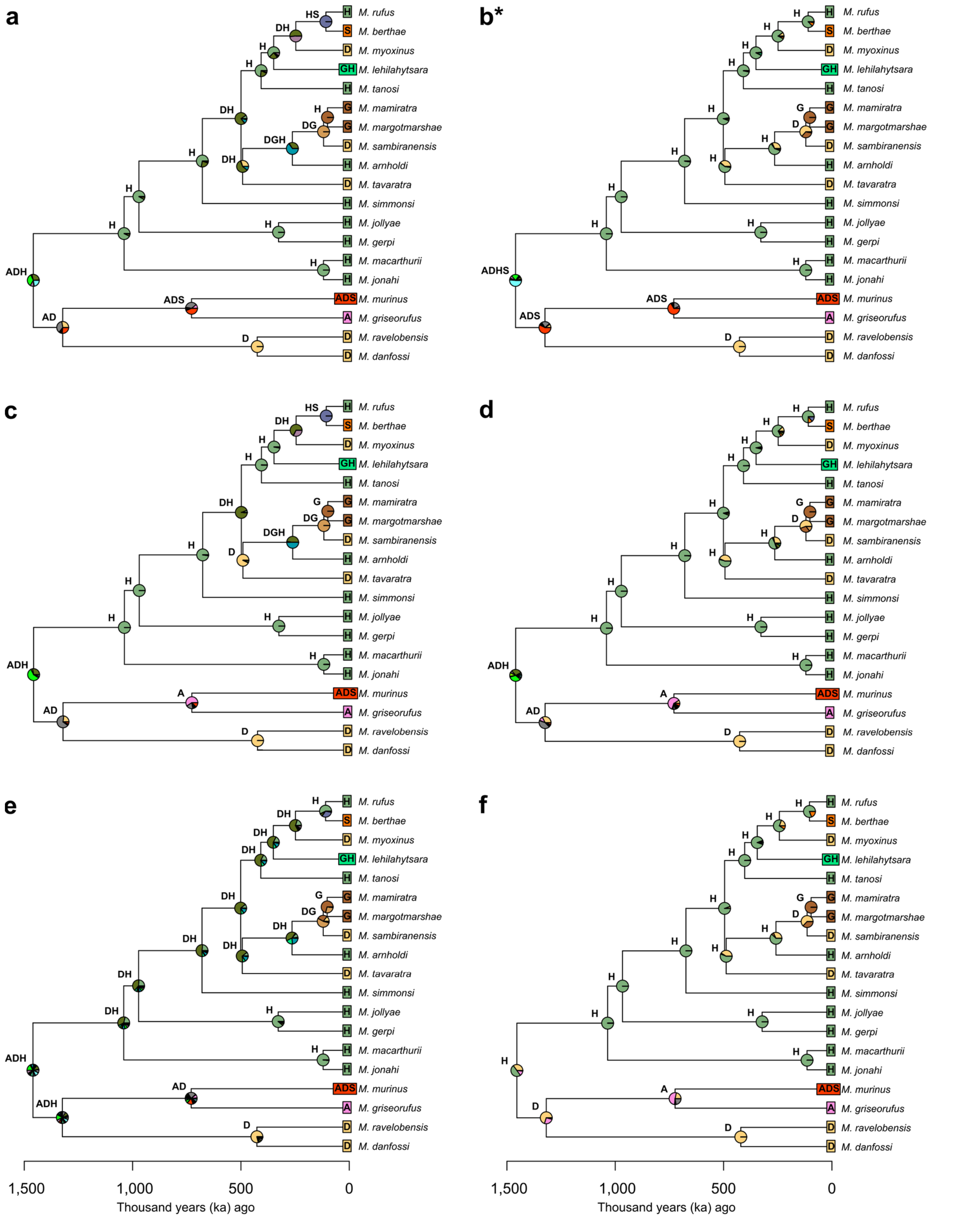

Supplementary Figure 16: Ancestral biogeographic regions of *Microcebus* lineages (A: arid spiny bush, D: dry deciduous forest, G: grassland/woodland mosaic, H: evergreen rainforest, S: succulent woodlands; see Yoder et al.[8]), estimated with BioGeoBears under the following models: DEC (**a**), DEC+J (**b**)**,** DIVA-like (**c**)**,** DIVA-like+J (**d**), BAYAREA-like (**e**), BAYAREA-like+J (**f**). Node letters indicate the most likely region. Multiple abbreviations at a single node refer to the combined region (e.g., DH: dry deciduous forest and evergreen rainforest). The best fitting model is indicated by an asterisk. Model details are given in Supplementary Table 8.

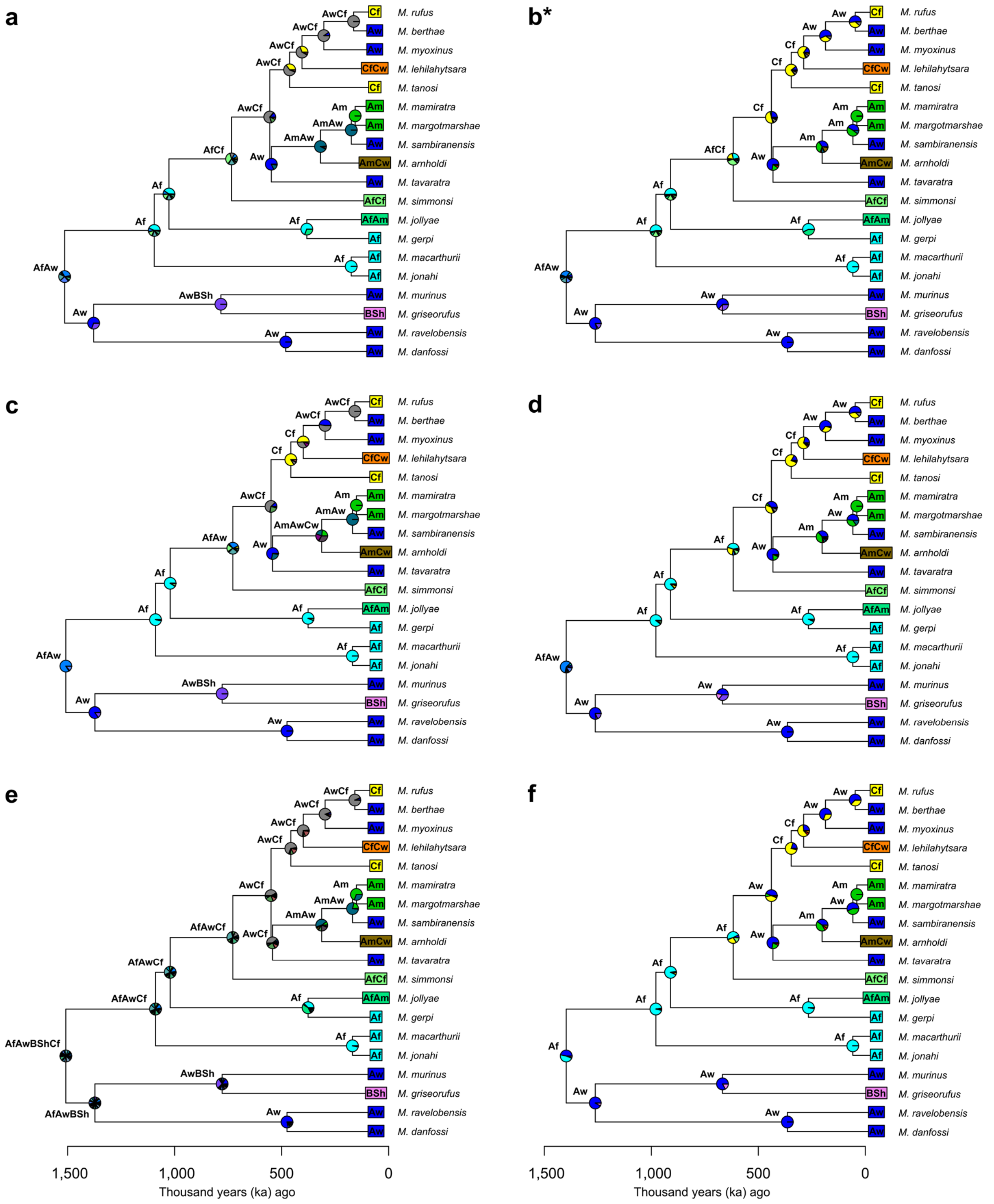

Supplementary Figure 17: Ancestral biogeographic regions of *Microcebus* lineages following the Köppen-Geiger climate classification (Af: tropical (rainforest), Am: tropical (monsoon), Aw: tropical (savannah, dry winter), BSh: dry (semi-arid or steppe, hot), Cf: temperate (no dry season), Cw: temperate (dry winter); see Beck et al., 2018[54]), estimated with BioGeoBears under the following models: DEC (**a**), DEC+J (**b**), DIVA-like (**c**), DIVA-like+J (**d**), BAYAREA-like (**e**), BAYAREA-like+J (**f**). Node letters indicate the most likely region. Multiple abbreviations at a single node refer to the combined region (e.g., AfCf: tropical and temperate). The best fitting model is indicated by an asterisk. Model details are given in Supplementary Table 8.

Morphological stasis and neutral climatic niche evolution (Supplementary Figs. 18 to 21)

To assess the power of the test statistic, i.e., Spearman's correlation coefficient (r_s) between node age and morphometric overlap, we carried out a cross-validation analysis on data simulated under either a Brownian motion (BM) or stationary Ornstein-Uhlenbeck (OU) model (see Methods). By considering only cases in which one of the two models could be uniquely identified (i.e., ignoring *Both-rej* and *none* in Supplementary Fig. 18ac), cross-validation analysis showed that data from a BM and OU model of evolution have a 97% and 91% probability, respectively, of rejecting the alternative model. It also revealed that the proposed simulation-based approach based on r_s has more power in distinguishing BM or OU models of evolution than AIC-based criteria of model selection (Supplementary Fig. 18bd).

Accordingly, our results show that the observed correlation between node age and morphometric overlap was more likely under a stabilising selection (stationary OU) than neutral random walk (BM) or early-burst (EB) model of evolution. However, an OU-like pattern can be generated also by more complex evolutionary processes[59], although this is also true for BM-like patterns[60]. Moreover, we acknowledge that an EB model may be difficult to detect with extant species alone[61,62], and therefore rejection of this model should be re-assessed, if fossil data would become available for the genus.

Nevertheless, we can use the stabilising selection model described by Lande[63] to evaluate whether the estimated parameters in the OU model can find reasonable justification in the genus *Microcebus*. In this model, the net rate of trait evolution over short and long time scales are expressed by $Var(\bar{z}) = \frac{h^2\sigma^2}{N_e}$ and $\alpha = \frac{(\omega^2+\sigma^2)}{2N_e}$, respectively[64,65], where h^2 is the trait heritability, ω^2 is the within-species trait variance, N_e is the effective population size and ω^2 is the strength of stabilising selection. Using morphological heritability estimates on captive *M. murinus* (head depth, head length, tibia length, tarsus length and birth weight; h^2=0.16–0.32[66]), average within-species trait variance estimated from our data, and considering that ω^2 typically ranges between 3 and 50 in the wild[67], we note that the α values estimated here (0.002–0.008) would require an average N_e between 220 and 1,300 across *Microcebus* species under relatively weak selection (ω^2 = 3), or between 3,000 and 15,000, under strong stabilising selection (ω^2 = 50). Similarly, assuming a generation time of 2.5 years[68], we estimate a net rate of trait evolution over short time scales ranging between 2.5 x 10^{-6} and 3.7 x 10^{-5} per generation. This implies an average N_e between 5,000 and 80,000. Following the rationale of Harmon et al.[69], our estimated rates of evolution at short and long time scales are compatible with typical N_e values in the wild[67] and in *Microcebus* species[16], suggesting that the single

optimum model (i.e., the OU model) could be a reasonable model for morphological evolution in this genus.

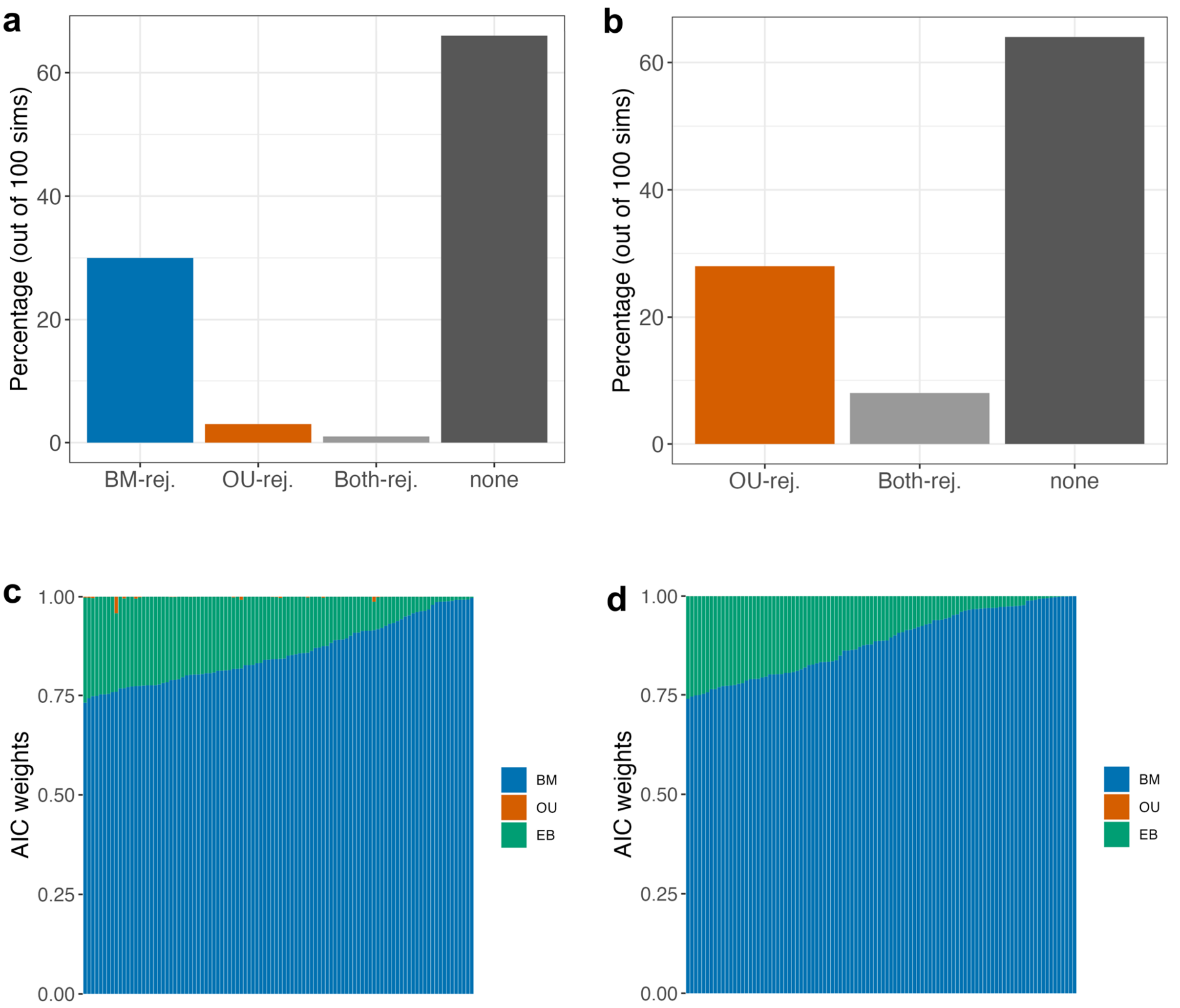

Supplementary Figure 18: Validation of the morphological stasis analysis and comparison with the AIC weights approach. **Top row:** Cross-validation analysis for Spearman's correlation coefficient (r_s) between node age and morphometric overlap. Since the fitted early burst (EB) model converged to a Brownian motion (BM) model, only BM and Ornstein-Uhlenbeck (OU) models were considered for cross-validation analysis. Proportion of model rejection for the 100 independent morphometric data sets simulated under the single optimum OU (**a**) and a BM model (**b**) (BM-rej: reject the BM model; OU-rej: reject the OU model; Both-rej: reject both BM and OU models; none: reject neither the BM nor the OU model). **Bottom row:** Proportion of AIC weights for the three models of morphometric evolution fitted to each of the 100 simulated morphometric data sets under the single optimum OU (**c**) and the BM (**d**) model. The results suggest that the proposed approach for model selection based on r_s is more accurate than using AIC weights.

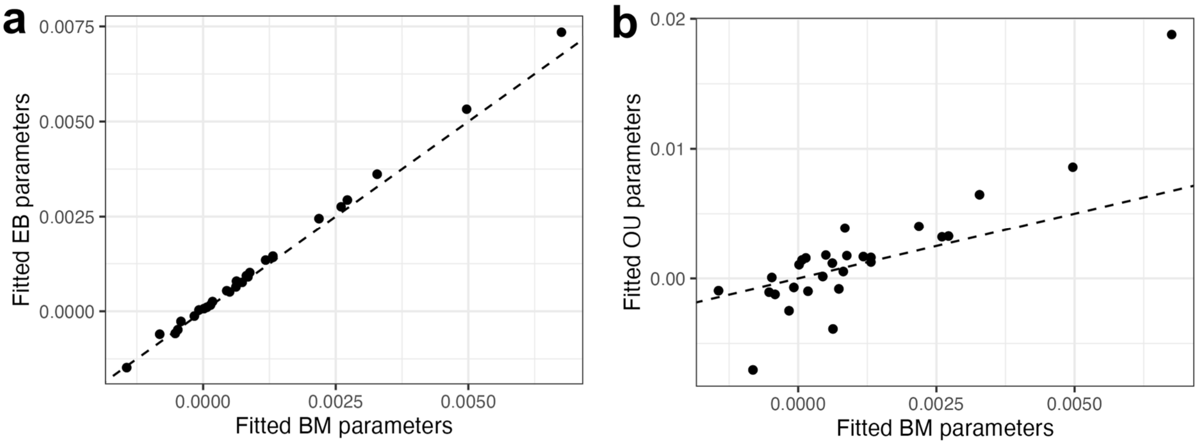

Supplementary Figure 19: Comparison of early-burst (EB), Brownian motion (BM) and Ornstein-Uhlenbeck (OU) models fitted to morphometric data. The parameters correspond to the net rates of trait evolution and trait pair covariation. **a,** Fitted parameters of the EB and BM models are nearly identical. **b,** In contrast, fitted parameters of the OU model differ from those of the BM model. These findings suggest that the fitted EB model converges to the BM model.

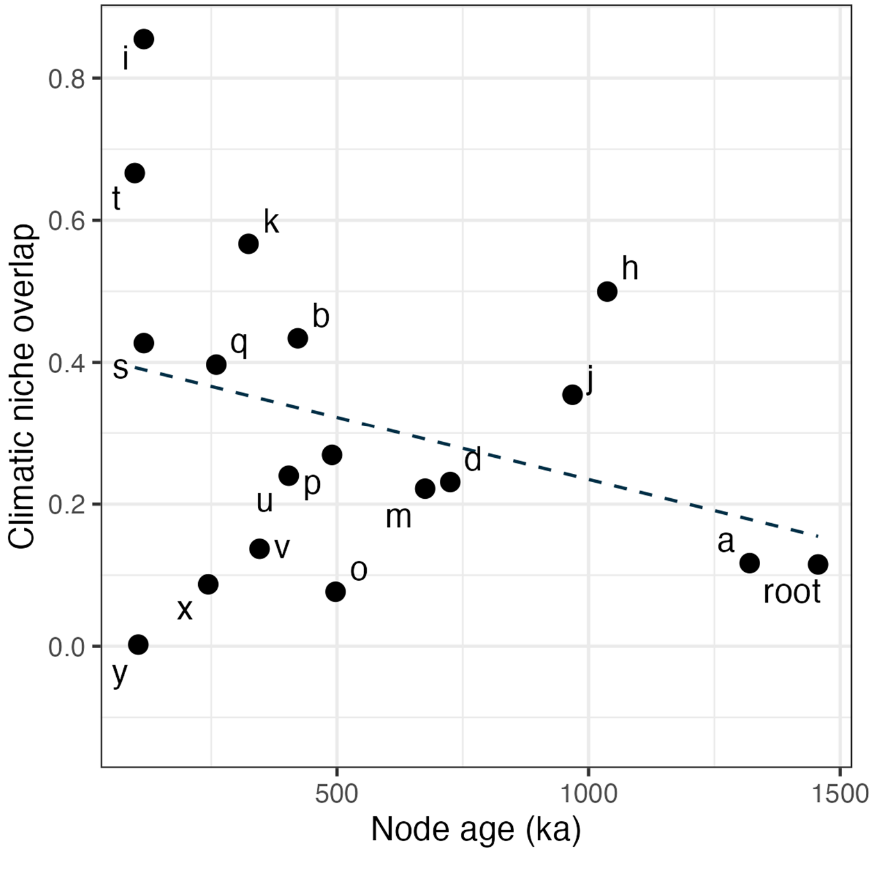

Supplementary Figure 20: Changes in climatic niche overlap along the *Microcebus* phylogeny, measured using Schoener's D. Letters refer to node labels in Fig. 2b. The correlation between node age and climatic niche overlap was not significant (Pearson's correlation test; r_s = -0.269; p = 0.281).

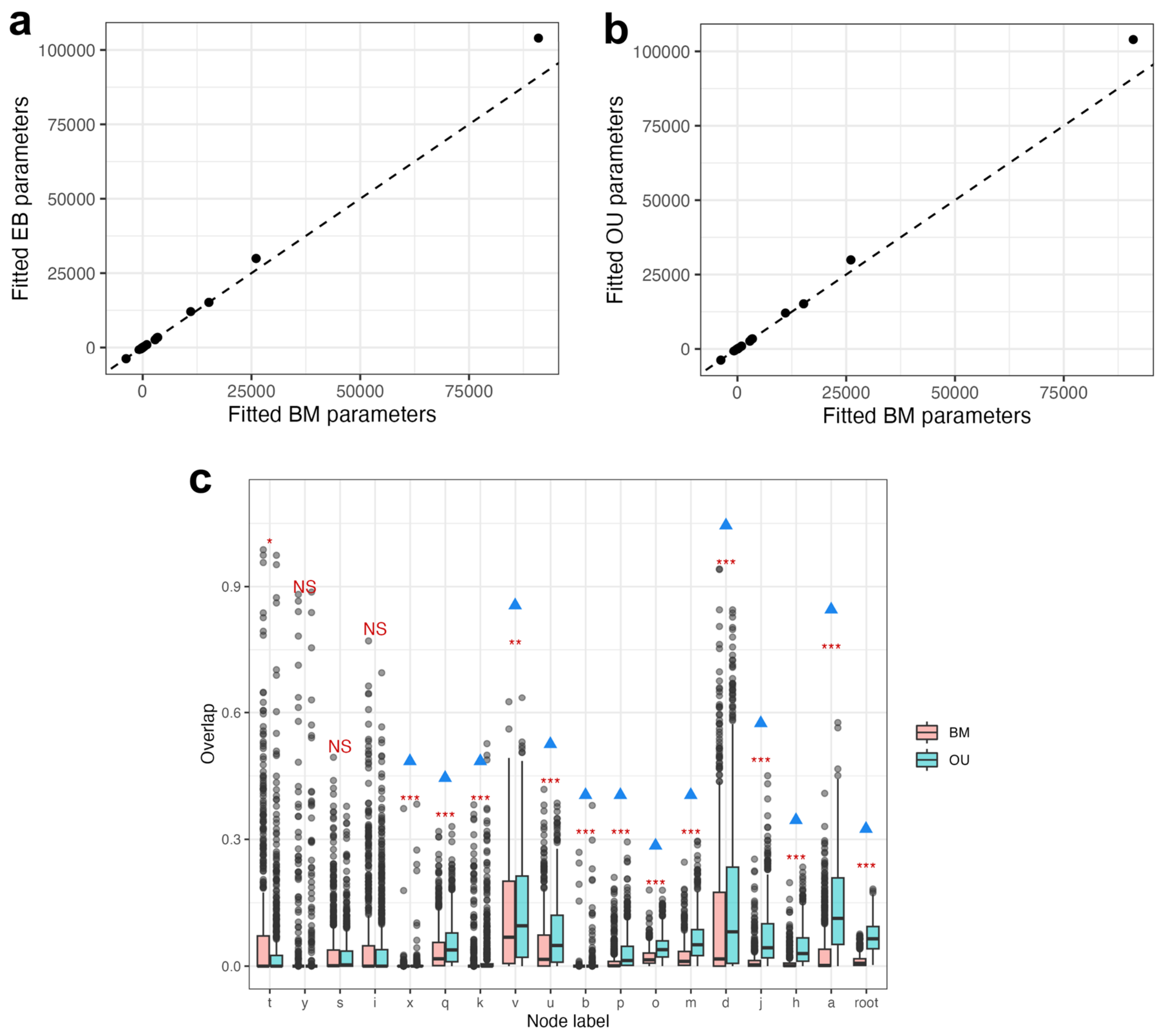

Supplementary Figure 21: Comparison of early-burst (EB), Brownian motion (BM) and Ornstein-Uhlenbeck (OU) models of climate niche evolution. **a** and **b**, Parameter estimates obtained after fitting the data to an EB and OU model, respectively. The parameters correspond to the net rates of trait evolution and trait pair covariation. The results show that fitted parameters of the EB and OU models are nearly identical with those of the BM model. **c**, Distribution of climatic niche overlap along nodes of the *Microcebus* phylogeny, obtained from simulations under the BM and OU models. Blue triangles indicate comparisons where the average overlap for a given node is significantly higher in the OU model compared to the BM model, which would be expected when both models show similar net rates of trait evolution. NS: not significant.

Change in conservation status

Following IUCN guidelines[70], we provide new conservation status recommendations for all valid *Microcebus* species after taxonomic revision on the basis of their extent of occurrence (EOO), area of occupancy (AOO) and loss of their AOO during the last 11.5 years (corresponding to approximately three generations), calculated using our extensive sampling. We propose to synonymise two microendemic Critically Endangered (CR; *M. manitatra*, *M. marohita*), three Endangered (EN; *M. bongolavensis*, *M. ganzhorni*, *M. mittermeieri*), one Data Deficient (DD; *M. boraha*) and one not yet evaluated (*M.* sp. 1) candidate species, resulting in a lower recommended level of endangerment (i.e., that of their respective senior synonym) for these previously assessed lineages (Figs. 2d; Supplementary Table 12). In addition, synonymising *M. bongolavensis* and *M. ravelobensis* would reduce the IUCN category of *M. ravelobensis* from Vulnerable to Near Threatened. The effect of increased sampling and the synonymisation of *M. mittermeieri* on the IUCN classification of *M. lehilahytsara* has already been formally estimated by Dolch et al.[71], which we confirm here. Additional sampling also led to a lower recommended level of endangerment for the five species *M. gerpi* (already suggested by Rakotondravony et al.[27]), *M. macarthurii*, *M. rufus, M. simmonsi* and *M. tanosi*. Conversely, our re-assessment does not indicate a necessity to raise the level of endangerment above the current IUCN classification for any species. It should be noted, however, that all 19 species lost significant portions of their AOO due to deforestation during the last three generations. The mean loss of habitat was estimated at 21.6% (SD = 12.4%), ranging from 7.8% for *M. tavaratra* to 58.2% for *M. jonahi* (Supplementary Table S12). The effects of forest degradation and fragmentation could not be conclusively assessed.

Supplementary methods

Sampling (Supplementary Fig. 22)

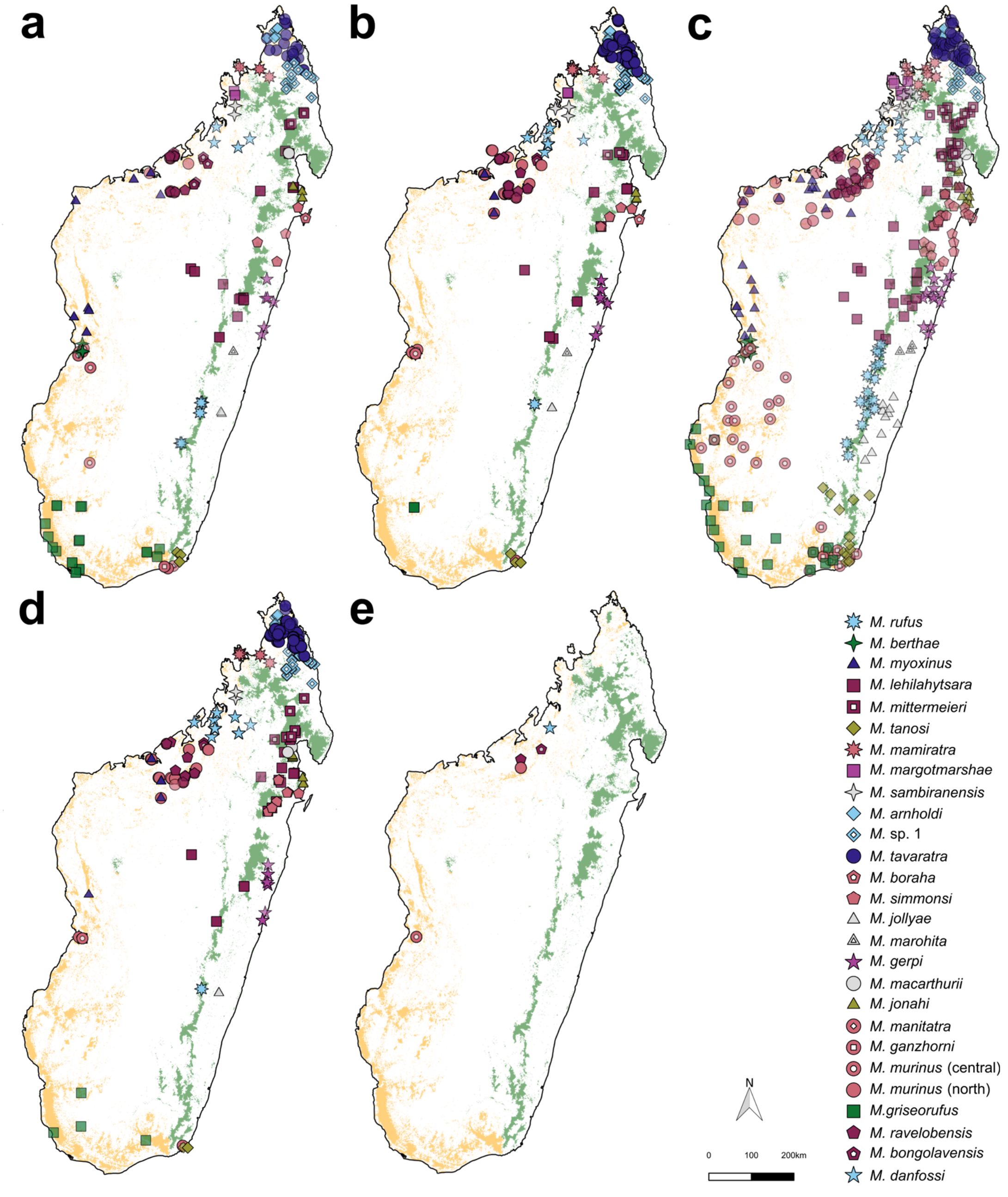

Supplementary Figure 22: Maps of *Microcebus* samples used in this study: **a,** RAD sequencing data. **b,** Morphometric data after filtering. **c,** Bioclimatic data after rarefaction. **d,** Data on reproductive activity produced in this study after filtering (additional records were added from the literature and are not illustrated here; see Supplementary Table S16). **e,** Acoustic data, which was complemented by data collected from individuals at the Institute of Zoology of the University of Veterinary Medicine Hannover, Foundation, which cluster genetically with *M. ganzhorni*. Note that coordinates for several samples are not available and therefore not plotted. Comprehensive sample lists can be found in Supplementary Tables 13 to 17.

Library preparation

RAD libraries were prepared following the three protocols described in Poelstra et al.[16] (detailed for each sample in Supplementary Table S18):

1. Oregon: Library preparation was based on Genomic Resources Development Consortium et al.[72]. Specifically, 40 – 100 ng of extracted genomic DNA were digested with the *SbfI* restriction enzyme (New England Biolabs) and subsequently ligated to the P1 adapters[73]. Up to 48 samples were pooled into sub-libraries and sheared for 5 min using a Bioruptor for 6 min to an average target size of 500 bp. Next, end-repair and 3' adenylation were performed, P2 adapters were ligated, and libraries were amplified in 14 cycles of PCR. Finally, sub-libraries were purified with AMPure XP beads (Agencourt), pooled based on yield and single-end sequenced (100 bp, 48 individuals / lane) on an Illumina HiSeq 2000 at the University of Oregon Core Facility.
2. Toulouse: Library preparation was also based on Genomic Resources Development Consortium et al.[72]. In contrast to the protocol mentioned above, 40 – 200 ng of genomic DNA were used, sub-libraries were sheared for 45 s in Covaris® M220, only 10 PCR cycles were conducted, and sequencing was performed on an Illumina HiSeq 3000 (paired-end, 150 bp, 96 individuals / lane) at the GenoToul Sequencing Platform Facility (Toulouse, France).
3. Idaho: Library preparation was based on Ali et al.[74]. Specifically, 50 ng of genomic DNA were digested with the *SbfI* restriction enzyme (New England Biolabs) and subsequently ligated to custom biotinylated and barcoded adapters. 48 samples were pooled and sheared with a Covaris® M220 to an average target size of 400 bp. Fragments were subsequently enriched with streptavidin beads, and libraries were prepared with the NEBNext Ultra DNA Library Prep Kit (New England Biolabs). Final libraries were paired-end sequenced (150 bp, 48 – 96 individuals / lane) on an Illumina HiSeq 4000 at the Vincent J. Coates Genomic Sequencing Laboratory of the University of California, Berkely, or at the Duke Center for Genomics and Computational Biology Sequencing Facility.

Species delimitation (Supplementary Figs. 23 to 27)

Genomics (isolation-by-distance):

The introduced statistical test quantifies patterns of IBD between versus within taxa, assessing whether genetic distances between individuals of candidate species deviate from a model of

intraspecific spatial genetic structure. To account for the genome-wide variation of the genealogical process, we quantified IBD across genomic regions by dividing genomic data into contiguous windows containing a fixed number of SNPs (Extended Data Fig. 1a). We visualised similarity in relatedness among windows using multidimensional scaling (MDS; Supplementary Fig. 24). The MDS plot was generated from a relatedness dissimilarity matrix among windows, given by the Euclidean distance in the relative position of each individual across windows as defined by the two main components of window-based PCAs. MDS was performed using the functions *eigen_windows*, *pc_dist* and *cmdscale* from the R package 'lostruct' v0.0.0.9000[75].

We used the normalised root mean square error (NRMSE) to quantify deviations of observed genetic distances between candidates from those predicted by the geographic clines in genetic distance within candidates (Extended Data Fig. 1bc). The NRMSE normalises genetic distances between and within candidates by the range of observed distances between candidates, thus facilitating comparisons among species complexes with different IBD scales within candidates. The predicted values were obtained from the linear regression model fitted to the within-taxon geographic and genetic distances. More specifically, given a genomic window *i*, pairwise comparisons within candidate 1 (n_1), within candidate 2 (n_2), and between candidates (n_3), we fitted a linear regression model for n_1 and n_2, separately:

$$y_1 = m_1x_1 + b_1 \text{ and } y_2 = m_2x_2 + b_2$$

where x_1 and x_2 are the pairwise geographic distances (natural logarithm) within candidate 1 and candidate 2, respectively, and y_1 and y_2 are the corresponding pairwise average number of nucleotide differences (π). m and b are the coefficients of the fitted models, which are estimated via a least-squares approach. For instance, for candidate 1, we would estimate:

$$m_1 = \frac{n_1(\sum x_1y_1) - (\sum x_1)(\sum y_1)}{n_1(\sum x_1^2) - (\sum y_1^2)^2} \text{ and } b_1 = \frac{\sum y_1 - m_1(\sum x_1)}{n_1}$$

Then, the predicted values of the pairwise genetic distances between candidates ($\widehat{y_3}$) were obtained from the corresponding observed pairwise geographic distances (x_3) using the following expressions:

$$\widehat{y_{3(1)}} = m_1x_3 + b_1 \text{ and } \widehat{y_{3(2)}} = m_2x_3 + b_2$$

Ultimately, we used the predicted ($\widehat{y_3}$) and observed (y_3) pairwise genetic distances between candidates for computing NRMSE:

$$NRMSE_1 = \frac{\sqrt{\frac{\sum_{n_3}\left(\widehat{y_{3(1)}} - y_3\right)^2}{n_3}}}{\left(y_3^{max} - y_3^{min}\right)}$$

The fitting of the linear regression model and prediction of genetic distances between candidates were performed with the R functions *lm* and *predict.lm*, respectively. Ultimately, we combined NRMSE estimates across all genomic windows to generate two NRMSE distributions for a given candidate pair, each obtained from the comparison with the intraspecific genetic diversity of one of the two candidates (Supplementary Fig. 25), which were then compared to the reference distributions (Extended Data Fig. 1d). While two reference distributions were obtained this way from *M. lehilahytsara* and *M. mittermeieri*, a different approach was used to generate the reference distribution from *M. tavaratra*. More specifically, genetic distances between individuals were classified into "between" and "within" (representing the majority of comparisons between fragmented and within continuous populations, respectively), using k-means clustering (Supplementary Fig. 23cd). To quantify the discontinuity in IBD that could be expected in a spatially structured yet interconnected species, the NRMSE was then calculated across genomic windows based on these two clusters, resulting in only one reference NRMSE distribution.

We consider *M. tavaratra* and *M. lehilahytsara* (incl. *M. mittermeieri*) appropriate reference systems because they are relatively widely distributed, comprise both larger continuous and smaller fragmented populations and are therefore hypothesised to exhibit comparably high intraspecific variation in taxonomic characters[16,18,21,40,76], that can serve as a an empirical null-model of variation to conservatively delimit species. In addition, their population genomic structure is well-characterised, with clear patterns of isolation-by-distance and/or gene flow between populations and no evidence for the presence of diverging lineages or potential candidates within these species (see Supplementary results and discussion: Species delimitation and diagnosis and Extended Data Fig. 3 for more information on *M. lehilahytsara* and *M. mittermeieri*, and Supplementary Fig. 23 for *M. tavaratra*). Although the plot of genetic vs. geographic distances in Supplementary Fig. 23c does indicate a discontinuity (i.e., gap between comparisons given in blue and red), higher genetic distances (red) are not attribuSupplementary Table only to two or few geographically separated populations, which we would expect if allopatric speciation explained the discontinuity. Rather, they represent comparisons between several forest patches at varying distances to each other (Supplementary Fig. 23d), even though we acknowledge that comparisons involving one of three sampling sites (named X, Y and Z) are overrepresented. Moreover, comparisons among these forest patches also provide several data points with lower genetic distances (i.e., blue data points are not only stemming from comparisons within forest patches), and this includes sites X, Y and Z. Taken together, the observed genetic structure in *M. tavaratra* can more likely be explained by stochastic processes and (recent) habitat fragmentation across the entire distribution of the species than by allopatric speciation between geographically isolated lineages.

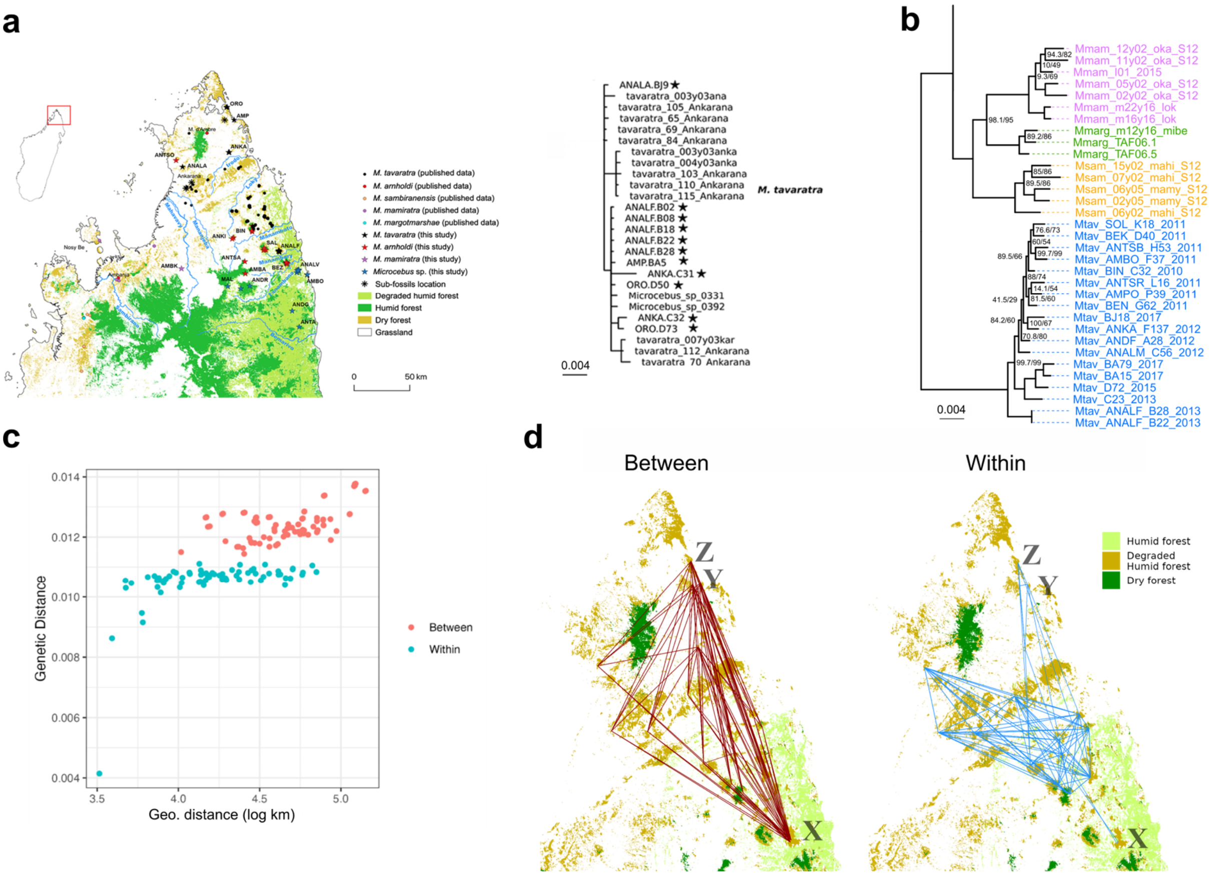

Supplementary Figure 23: Population genetic structure of *M. tavaratra*. **a,** Map of the species distribution across forest fragments (left) and phylogeny inferred from mtDNA indicating no major clusters (right; scale is substitutions per site); reproduced with permission of John Wiley & Sons, Inc. from Sgarlata et al.[21]. **b,** Maximum likelihood inference conducted in this work (5% maximum missing data) does not indicate major clusters either (node labels represent percent SH-aLRT/ultrafast bootstrap support if below 100; scale is substitutions per site), unlike in other candidate groups that are separated by comparably long branches (e.g., *M. mamiratra*, *M. margotmarshe* and *M. sambiranensis*). **c,** Genetic distances tend to increase linearly with the log of geographic distances (isolation-by-distance; IBD). Labelling of comparisons into “between” and “within” was done via k-means clustering of genetic distances and used for the NRMSE IBD analysis (see Methods). **d,** Geographic representation of data points in panel c (pairwise comparisons labelled as “between” and “within” are connected by red and blue edges, respectively). Although three sampling sites (named X, Y and Z) account for the majority of comparisons labelled as “between”, the latter are not restricted to these sites but involve several additional forest patches. Conversely, comparisons among these forest patches also provide several data points labelled as “within”, including the sites X, Y and Z. This does not indicate allopatric speciation between geographically isolated lineages or the presence of candidate species in *M. tavaratra*.

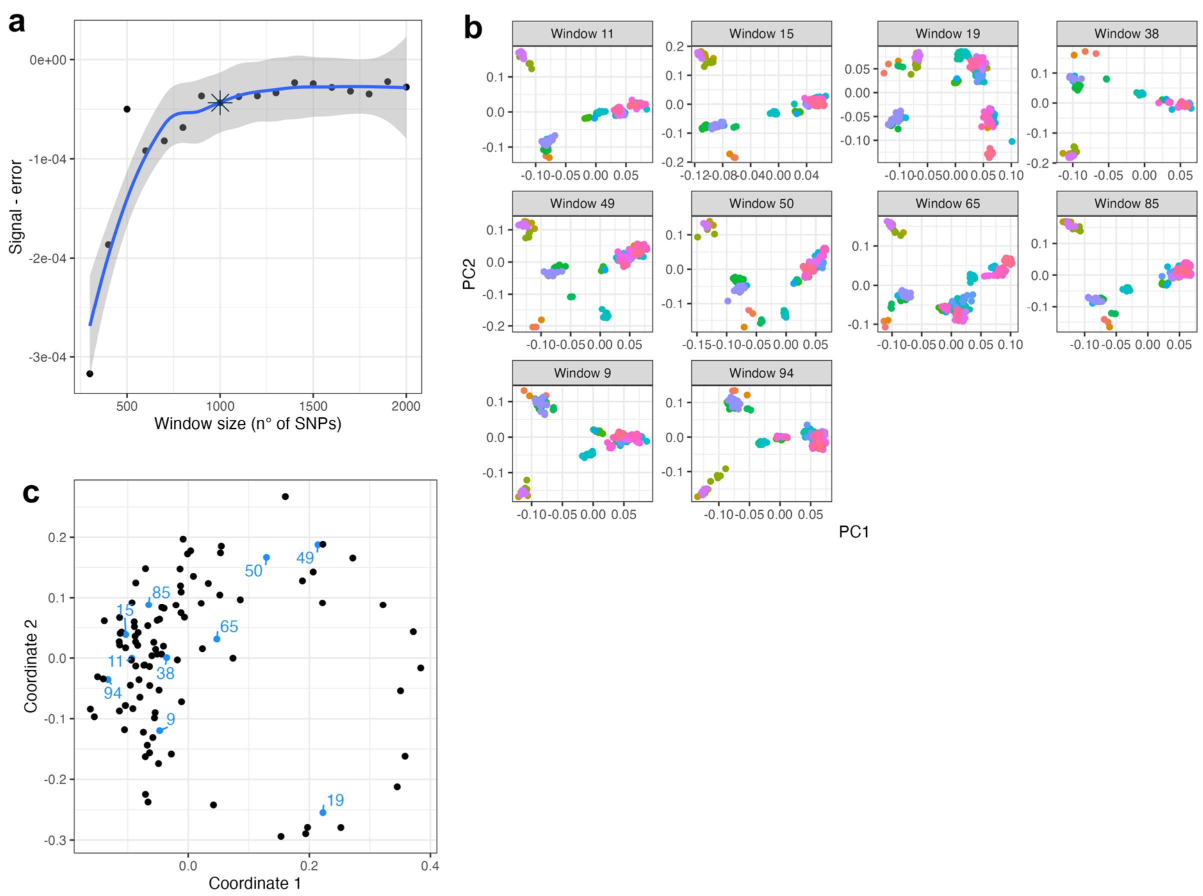

Supplementary Figure 24: Window-size selection and multidimensional scaling on genomic windows-based PCA. **a,** Changes in signal - error ratio with genomic window size, reaching a plateau at about 1,000 SNPs. Grey shading indicates 95% confidence interval. **b,** Multidimensional scaling of PCAs computed across genomic windows of 1,000 SNPs. Labels correspond to ten randomly selected windows. **c,** PCAs of the ten randomly selected windows shown in panel b.

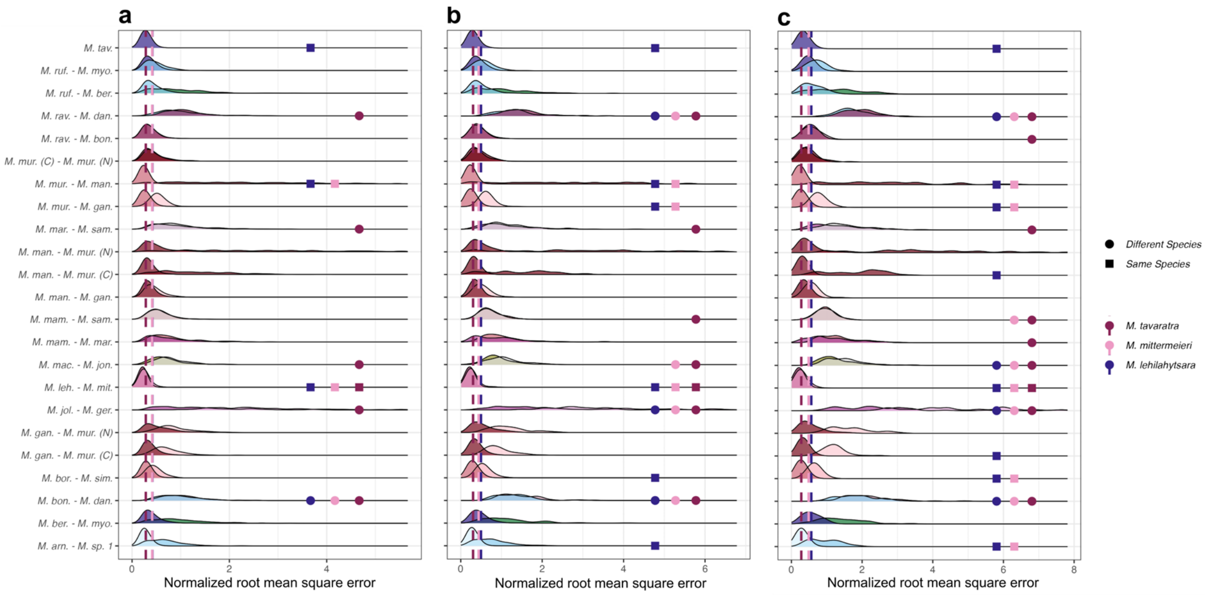

Supplementary Figure 25: Normalised root mean square error (NRMSE) distributions of within and between candidate isolation-by-distance (IBD) across *Microcebus* candidate species pairs and different window sizes: **a,** 500 SNPs. **b,** 1,000 SNPs. **c,** 2,000 SNPs. Taxon names refer to the first three letters of the candidate species epithet. For each pair, two distributions are shown, as the NRMSE has been calculated with respect to intraspecific patterns of IBD in each taxon. Vertical dashed lines correspond to 0.95 quantiles of *M. tavaratra*, *M. lehilahytsara* and *M. mittermeieri* NRMSE distributions, which were used as thresholds for species delimitation (see Methods in main text).

Genomics (genealogical divergence index):

While we followed Jackson et al.[77] in using a *gdi* of 0.2 as a threshold for synonymisation, we urge caution as this value likely underestimates the minimum *gdi* for mammalian species differentiation. Jackson et al. offer a single rule of thumb for all taxa despite large variation in *gdi* across orders. Mammals generally seem to exhibit higher *gdi* than birds and insects. In addition, although the upper threshold value is relatively well justified in Jackson et al. (i.e., “the upper quartile range of *gdi* values observed for groups identified as ‘populations’ in the 178 empirical datasets never rises above 0.66, suggesting that a *gdi* value above ~0.7 signals that speciation has likely occurred”), the lower estimate lies far from its justified value (i.e., “two species are never inferred with high AIC weight when the *gdi* is below ~0.3”). Useful rules of thumb should be tailored to specific taxonomic groups based on their *gdi* distribution. A one-size-fits-all approach is prone to misinterpretations. Therefore, we advocate further research to estimate a more appropriate *gdi* threshold for synonymisation of mammal taxa, which will

aid distinguishing intraspecific lineages with limited divergence from those undergoing speciation.

Reproductive activity:

Differentiation in reproductive activity can be a key factor of speciation. In many cheirogaleid species, reproductive activity is highly synchronised, with slight temporal shifts potentially causing reproductive isolation[78]. We therefore assembled 2,354 presence/absence records of oestrus, pregnancy or lactation in females and of enlarged testes in males at the time of capture (1,006 male and 1,348 female records across 24 described *Microcebus* species) from our own research and the literature (Supplementary Table 16). For literature records without specific dates of assessment, the time period mentioned (e.g., first half of October) was covered in five-day steps to represent assessments of that period as an approximation (e.g., October 5th and October 10th as approximated dates of assessment). These data partly included re-assessments of the same individuals across a longer study period, but also singular assessments at the day of capture without later recapture. Following Rina Evasoa et al.[19], the presence of oestrus, pregnancy or lactation in females and the presence of enlarged testes in males were used as reproductive indicators. For each candidate species and month of the year, we estimated the proportion of reproductively active individuals (i.e., in oestrus for females or with enlarged testes for males) and total individuals surveyed. Because lactation and pregnancy can be diagnosed about 2 – 3.5 months and two months after oestrus, respectively[79,80], the corresponding dates were adjusted to obtain the approximate timing of oestrus by subtracting 2 – 3.5 months and 1 – 2 months, respectively (effects of the adjustment method on inferred reproductive activity are illustrated in Supplementary Fig. 26). Confidence intervals were estimated using Wilson's method, implemented in the function *binom.confint* of the R package 'binom' v1.1-1.1[81].

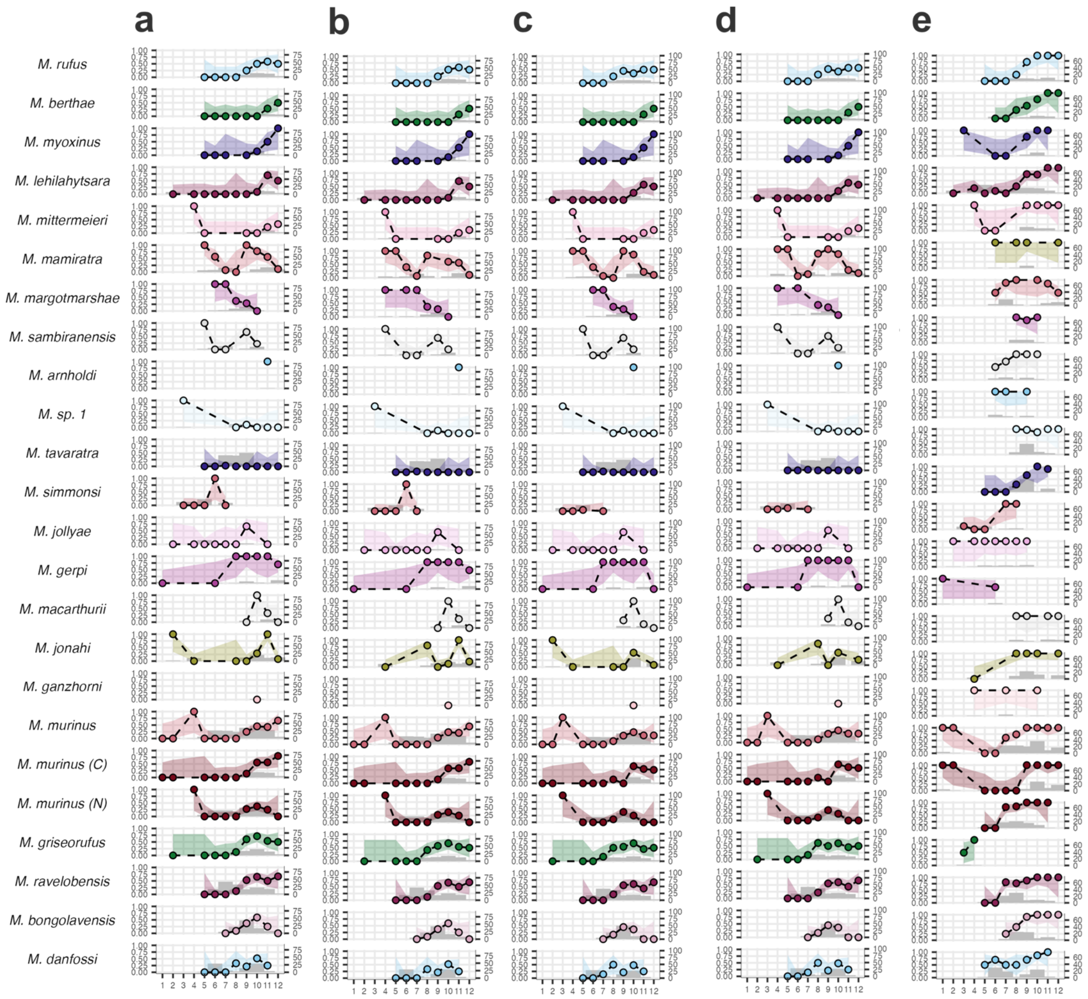

Supplementary Figure 26: Female and male reproductive schedules across *Microcebus* candidate species. **a,** Females with adjustment of 2 months for lactation and 1 month for pregnancy. **b,** Females with adjustment of 3.5 months for lactation and 1 month for pregnancy. **c,** Females with adjustment of 2 months for lactation and 2 months for pregnancy. **d,** Females with adjustment of 3.5 months for lactation and 2 months for pregnancy. **e,** Male reproductive schedule. x-axis: month (1 – 12). y-axis: proportion of reproductive individuals (females: oestrous; males: with enlarged testes). Grey histograms indicate sample size. Coloured polygons delimit the confidence interval around the proportion of reproductive individuals according to Wilson's method.

Acoustic communication:

Acoustic communication can be crucial for species recognition or mate choice. Accordingly, bioacoustic tools have already been used in diverse animal species to study or clarify taxonomic questions (e.g., insects[82], anurans[83], mammals[84,85]). Therefore, we retrieved acoustic data from the sound archive of the Institute of Zoology of the University of Veterinary Medicine Hannover, Foundation for two clades, comprising the candidate species *M. bongolavensis*, *M. danfossi* and *M. ravelobensis* (alert calls) and *M. ganzhorni*, and *M. murinus*

(advertisement calls), respectively. Data were obtained during behavioural studies at different locations in Madagascar and in the captive *Microcebus* breeding colony of the Institute of Zoology, which clusters genetically with *M. ganzhorni* (see Mmur_Rhodos_S12 and Mmur_Gina_S12 in Supplementary Figs. 1 to 5). The housing conditions of the breeding colony were regularly licensed and proved by the local veterinary authorities (licence no.: 42502/1TiHo). Depending on the respective legislation for the year of recordings, studies were performed in accordance with the law of the European Community regulations on the protection of experimental animals and the guidelines of the German Animal Welfare Act and approved by the Niedersächsisches Landesamt für Verbraucherschutz und Lebensmittelsicherheit (LAVES), Germany (licence no.: AZ33. 19-42502-11A117; 33.12-42502-04-14/1454). Details on sampling locations, recording conditions and sample sizes are summarised in Supplementary Table 17.

Acoustic measurements were performed in PRAAT v5.4.0.4[86] using custom scripts. Since different call types were available for the two clades (i.e., alert vs. advertisement calls), acoustic parameters and settings were adapted to the respective acoustic structure (Supplementary Table 23). Alert calls (Tsak) are harmonic up- and down modulated calls produced in series of up to 100 calls (Supplementary Fig. 7). To avoid pseudoreplication in this kind of analysis, a maximum of two Tsak calls per series was selected. Tsak calls were band-pass filtered (75 – 60,000 Hz) and, for each call, eight acoustic parameters were measured (Supplementary Tables 23 and 24; see also Hasiniaina et al.[32] for details). Advertisement calls (Trill) are complex modulated sounds which consist of bouts of up to 30 syllables which differ in their frequency contour with the first syllable showing the highest variability (Supplementary Fig. 7). Trill calls were recorded with a Nagra IV-SJ tape recorder, which can only record calls up to 48 kHz. We therefore resampled all calls to the same sampling frequency of 96 kHz and conducted a band-pass filtering with a range of 9 – 45 kHz. We measured eight acoustic parameters characterising the first syllable of the call and four additional parameters characterising the whole call (Supplementary Tables 23 and 24). The available calls were recorded with different systems (e.g., analogue versus digital; different microphone sensitivities), which could hamper analytical power. To test for disturbing effects, we compared total duration and maximum fundamental frequency between the analogue and digital recording equipment for *M. murinus* (north) using linear mixed effect models which controlled for repeated measurement of the same individuals. Both parameters did not differ significantly between recording equipment ($t \leq 0.540$, $p \geq 0.598$). Visual inspections of the boxplots showed that vocalisations of the same location but recorded with different equipment were more similar (*M. murinus* (north): analogue versus digital) than vocalisations recorded in different locations using the same equipment (analogue: *M. murinus* (north) versus *M. murinus* (central); digital: *M. murinus* (north) versus *M. ganzhorni*; Supplementary Fig. 27), suggesting

that measurement bias had a negligible effect on our analysis. We therefore pooled all vocalisations per location for further analyses.

Following the procedure of morphological and climatic niche analyses, we constructed *n*-dimensional hypervolumes from the different call parameters and measured the maximum value of asymmetric overlap between sister candidate species[87,88] (Supplementary Table S25). To account for multicollinearity, we performed PCA beforehand and used the resulting PCs as input variables to the calculation of hypervolumes.

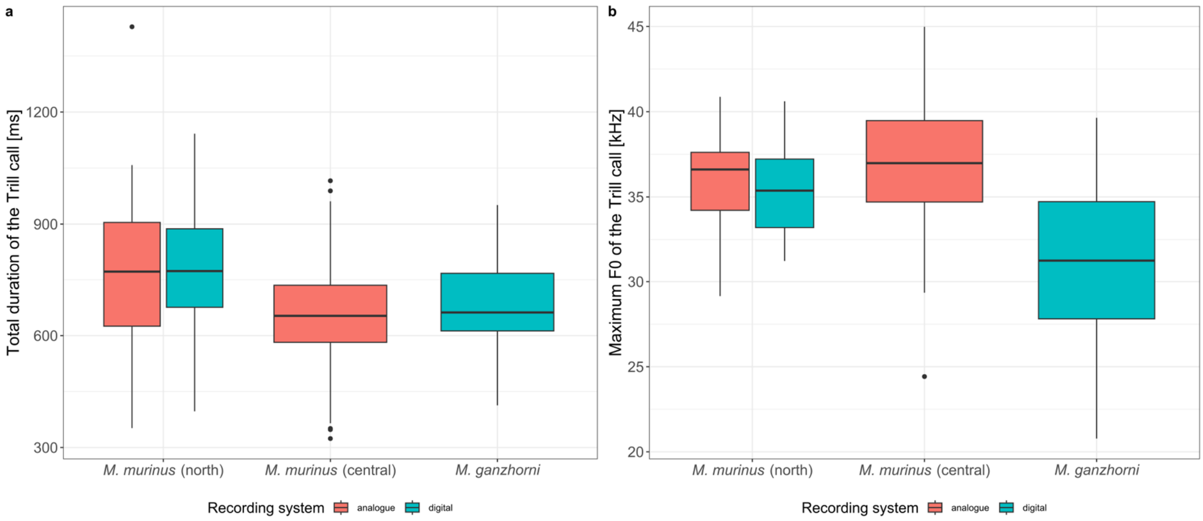

Supplementary Figure 27: Boxplots of the total duration (**a**) and the maximum fundamental frequency F0 (**b**) of Trill calls measured by different recording systems in the candidates *M. murinus* (north), *M. murinus* (central) and *M. ganzhorni* (n = 91 for each taxon; *M. murinus* (north): $n_{analogue}$ = 64 and $n_{digital}$ = 27). Box plots show the interquartile range (coloured boxes) with the median (black line) and quartiles plus 1.5 times the interquartile range (whiskers). Data points outside this range (outliers) are represented by black dots.

Divergence time estimation

While external evidence such as fossils is considered the gold standard for calibrating evolutionary distances in substitutions per site to substitutions per absolute time units, such calibrations are not available for the genus *Microcebus*, Lemuriformes, or older primate divergences. Given that only external calibrations are available for the sister group Lorisiformes and the nearest crown group, calibration is not available until Euarchontoglires (see reviews of evidence in Appendix 1 of dos Reis et al.[42] and Supplementary Table S1 of dos Reis et al.[89]). In addition, because of the recent divergences among *Microcebus* species evident from their genetic distances and previous studies[8,16], there is considerable risk that

conventional analyses with clock models would be compromised by biases towards older calibrations[43], on top of the technical biases that could be introduced by combining RADseq data with published genome assemblies. There is also the reasonable expectation that ignoring the coalescent process for the genus *Microcebus*, where internal branch lengths between speciation events are short, would overestimate the species split times[45]. Therefore, we applied a strategy to estimate divergence times that avoids the biases of much older external calibrations and concatenation by accounting for incomplete lineage sorting with the MSC model and transforming branch lengths from substitutions per site to substitutions per year based on external evidence from per-generation *de novo* primate mutation rates and *Microcebus* generation times.

Following Poelstra et al.[16], we used a mutation rate of 1.236×10^{-8} per site per generation and a generation time of 3.5 years to convert τ to years. To explore how uncertainty in these estimates affects inferred divergence times, we also did the conversion using a gamma distribution with a mean of 1.236×10^{-8} and a variance of 0.107×10^{-8}, as well as a lognormal distribution with a mean of ln(3.5) and a standard deviation of ln(1.16) for mutation rate and generation time, respectively. The mutation rate distribution is based on the mean of estimates found in different primates while roughly capturing their variance[90–96]. We did not use the point estimate of 1.52×10^{-8} per site per generation given for *Microcebus murinus* in Tiley et al.[90] because it might be inflated[96] and there is likely variation in germline mutation rates among individuals within a species and over time[97]. For generation times, data are much more difficult to gather, with only two studies at the time of writing that provide estimates from wild populations[68,98]. The lognormal distribution was constructed to be centred on the midpoint of the means from both studies (3.5 years), with variance adjusted to encompass the range. A lognormal distribution was chosen as we assume a skew such that more individuals on the early end of reproductive maturity are contributing to the population than older individuals. It ultimately assumes a time to reproduction of about two years and that few individuals reproduce beyond six years.

Modelling morphological and climatic niche evolution (Supplementary Figs. 28 to 29)

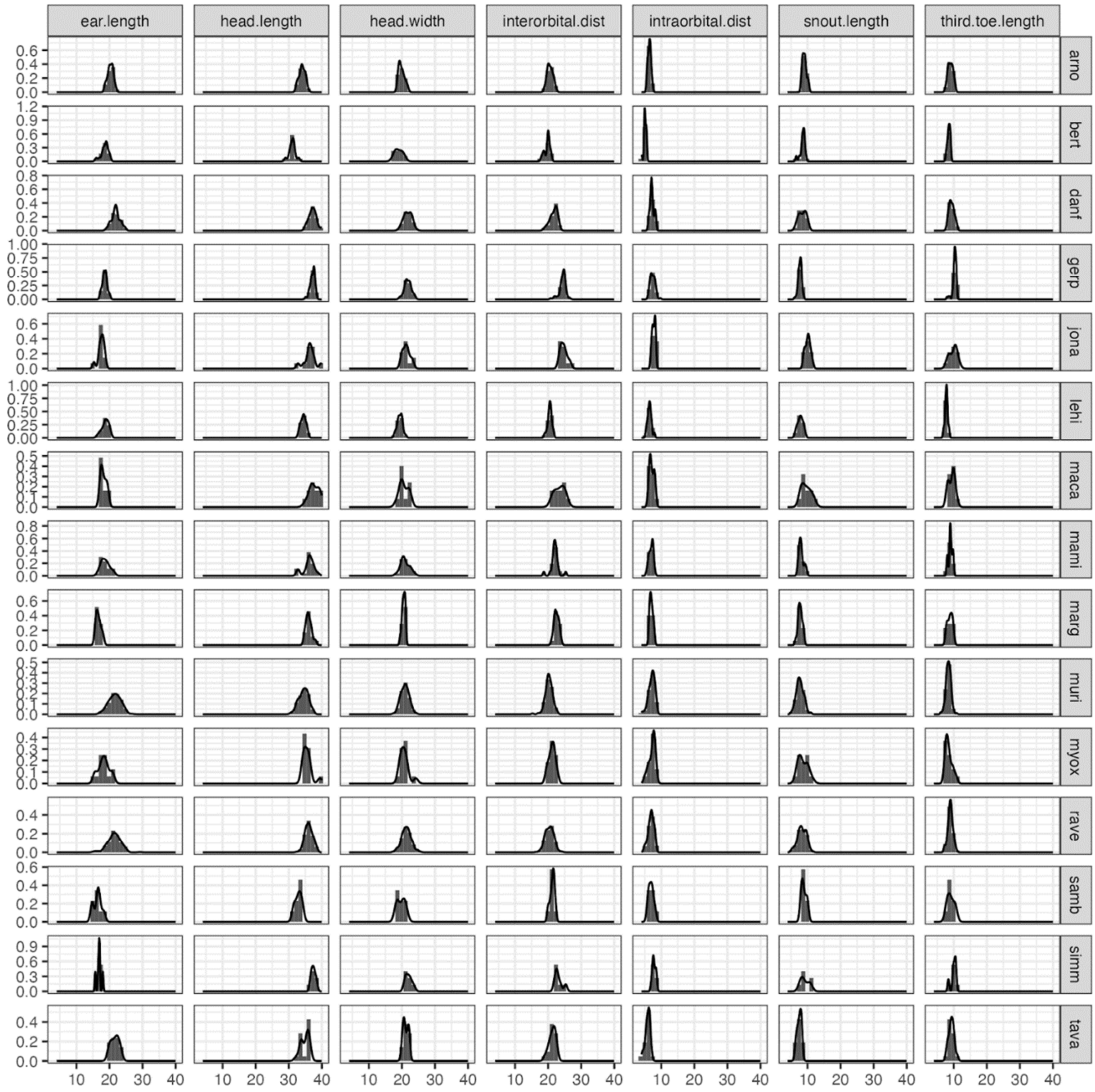

Supplementary Figure 28: Empirical distribution of each morphometric variable and species in the dataset with seven variables. Row names refer to the first four letters of the candidate species epithet.

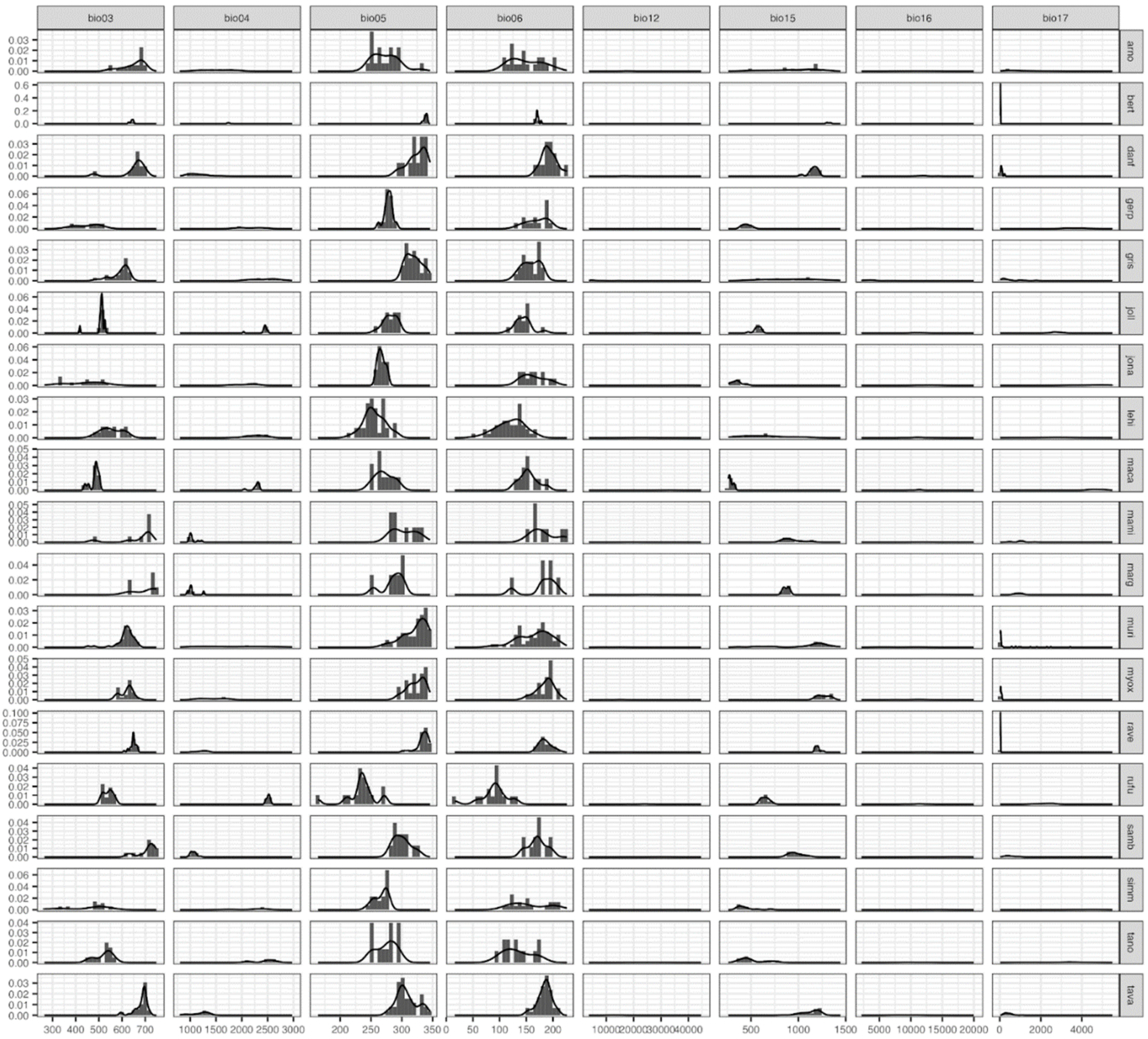

Supplementary Figure 29: Empirical distribution of each bioclimatic variable and species. Row names refer to the first four letters of the candidate species epithet. bio03: isothermality; bio04: temperature seasonality; bio05: maximum temperature of warmest month; bio06: minimum temperature of coldest month; bio12: annual precipitation; bio15: precipitation seasonality; bio16: precipitation of wettest quarter; bio17: precipitation of driest quarter.

References

1. Yoder, A. D. *et al.* Remarkable species diversity in Malagasy mouse lemurs (Primates, *Microcebus*). *Proc. Natl. Acad. Sci.* **97**, 11325–11330 (2000).
2. Louis, E. E. *et al.* Revision of the mouse lemurs (*Microcebus*) of eastern Madagascar. *Int. J. Primatol.* **27**, 347–389 (2006).
3. Louis, E. E. *et al.* Revision of the mouse lemurs, *Microcebus* (Primates, Lemuriformes), of northern and northwestern Madagascar with descriptions of two new species at Montagne d'Ambre National Park and Antafondro Classified Forest. *Primate Conserv.* **23**, 19–38 (2008).
4. Weisrock, D. W. *et al.* Delimiting Species without nuclear monophyly in Madagascar's mouse lemurs. *PLoS One* **5**, e9883 (2010).
5. Lei, R. *et al.* PhyloMarker – a tool for mining phylogenetic markers through genome comparison: Application of the mouse lemur (genus *Microcebus*) phylogeny. *Evol. Bioinforma.* **8**, EBO.S9886 (2012).
6. Weisrock, D. W. *et al.* Concatenation and concordance in the reconstruction of mouse lemur phylogeny: An empirical demonstration of the effect of allele sampling in phylogenetics. *Mol. Biol. Evol.* **29**, 1615–1630 (2012).
7. Louis, E. E. & Lei, R. Mitogenomics of the family Cheirogaleidae and relationships to taxonomy and biogeography in Madagascar. in *The Dwarf and Mouse Lemurs of Madagascar: Biology, Behavior and Conservation Biogeography of the Cheirogaleidae* (eds. Lehman, S. M., Radespiel, U. & Zimmermann, E.) 54–93 (Cambridge University Press, 2016).
8. Yoder, A. D. *et al.* Geogenetic patterns in mouse lemurs (genus *Microcebus*) reveal the ghosts of Madagascar's forests past. *Proc. Natl. Acad. Sci.* **113**, 8049–8056 (2016).
9. Everson, K. M. *et al.* Not one, but multiple radiations underlie the biodiversity of Madagascar's endangered lemurs. *bioRxiv* 2023.04.26.537867 (2023) doi:10.1101/2023.04.26.537867.
10. Fauskee, B., Crowl, A. A., Piatkowski, B., Yoder, A. D. & Tiley, G. P. Ancient introgression in mouse lemurs (Microcebus:Cheirogaleidae) explains 20 years of phylogenetic uncertainty. *Bull. Soc. Syst. Biol.* **3** (2024).
11. Peters, W. C. H. *Naturwissenschaftliche Reise nach Mozambique auf Befehl seiner*

Majestät des Königs Friedrich Wilhelm IV in den Jahren 1842 bis 1848 ausgeführt. Zoologie. I. Säugethiere (G. Reimer, 1852).

12. Saint-Hilaire, G. *Cours de l'histoire naturelle des mammifères*. (Pichon et Didiér, 1834).

13. Rasoloarison, R. M., Goodman, S. M. & Ganzhorn, J. U. Taxonomic revision of mouse lemurs (*Microcebus*) in the western portions of Madagascar. *Int. J. Primatol.* **21**, 963–1019 (2000).

14. Kappeler, P. M., Markolf, M., Rasoloarison, R. M., Fichtel, C. & Durbin, J. Complex social and political factors threaten the world's smallest primate with extinction. *Conserv. Sci. Pract.* **4**, e12776 (2022).

15. Roos, C. & Kappeler, P. Distribution and conservation status of two newly described cheirogaleid species, *Mirza zaza* and *Microcebus lehilahytsara*. *Primate Conserv.* **2006**, 51–53 (2006).

16. Poelstra, J. W. *et al.* Cryptic patterns of speciation in cryptic primates: Microendemic mouse lemurs and the multispecies coalescent. *Syst. Biol.* **70**, 203–218 (2021).

17. Schüßler, D. *et al.* Ecology and morphology of mouse lemurs (*Microcebus* spp.) in a hotspot of microendemism in northeastern Madagascar, with the description of a new species. *Am. J. Primatol.* **82**, e23180 (2020).

18. Tiley, G. P. *et al.* Population genomic structure in Goodman's mouse lemur reveals long-standing separation of Madagascar's Central Highlands and eastern rainforests. *Mol. Ecol.* **31**, 4901–4918 (2022).

19. Rina Evasoa, M. *et al.* Variation in reproduction of the smallest-bodied primate radiation, the mouse lemurs (*Microcebus* spp.): A synopsis. *Am. J. Primatol.* **80**, e22874 (2018).

20. Andriantompohavana, R. *et al.* Mouse lemurs of northwestern Madagascar with a description of a new species at Lokobe Special Reserve. *Occas. Pap. Museum Texas Tech Univ.* **259**, 1–23 (2006).

21. Sgarlata, G. M. *et al.* Genetic and morphological diversity of mouse lemurs (*Microcebus* spp.) in northern Madagascar: The discovery of a putative new species? *Am. J. Primatol.* **81**, e23070 (2019).

22. Hotaling, S. *et al.* Species discovery and validation in a cryptic radiation of endangered primates: Coalescent-based species delimitation in Madagascar's mouse lemurs. *Mol. Ecol.* **25**, 2029–2045 (2016).

23. Sukumaran, J. & Knowles, L. L. Multispecies coalescent delimits structure, not species. *Proc. Natl. Acad. Sci.* **114**, 1607–1611 (2017).

24. Leaché, A. D., Zhu, T., Rannala, B. & Yang, Z. The spectre of too many species. *Syst. Biol.* **68**, 168–181 (2019).

25. Radespiel, U. *et al.* First indications of a highland specialist among mouse lemurs (*Microcebus* spp.) and evidence for a new mouse lemur species from eastern Madagascar. *Primates* **53**, 157–170 (2012).

26. Rasoloarison, R. M., Weisrock, D. W., Yoder, A. D., Rakotondravony, D. & Kappeler, P. M. Two new species of mouse lemurs (Cheirogaleidae: *Microcebus*) from eastern Madagascar. *Int. J. Primatol.* **34**, 455–469 (2013).

27. Rakotondravony, R., Schüßler, D., Rovanirina, V. S. T., Ratsimbazafy, J. & Radespiel, U. Variation in abundance and habitat use of the critically endangered *Microcebus gerpi* across its fragmented range. *Am. J. Primatol.* **85**, e23553 (2023).

28. van Elst, T. *et al.* Diversification processes in Gerp's mouse lemur demonstrate the importance of rivers and altitude as biogeographic barriers in Madagascar's humid rainforests. *Ecol. Evol.* **13**, e10254 (2023).

29. Radespiel, U. *et al.* Exceptional diversity of mouse lemurs (*Microcebus* spp.) in the Makira region with the description of one new species. *Am. J. Primatol.* **70**, 1033–1046 (2008).

30. Miller, J. F. *Various subjects of natural history wherein are delineated birds, animals, and many curious plants: With the fructification of each plant, all of which are drawn and coloured from nature*. (1777).

31. Braune, P., Schmidt, S. & Zimmermann, E. Acoustic divergence in the communication of cryptic species of nocturnal primates (*Microcebus* spp.). *BMC Biol.* **6**, 19 (2008).

32. Hasiniaina, A. F. *et al.* High frequency/ultrasonic communication in a critically endangered nocturnal primate, Claire's mouse lemur (*Microcebus mamiratra*). *Am. J. Primatol.* **80**, e22866 (2018).

33. Zimmermann, E., Cepok, S., Rakotoarison, N., Zietemann, V. & Radespiel, U. Sympatric mouse lemurs in north-west Madagascar: A new rufous mouse lemur species (*Microcebus ravelobensis*). *Folia Primatol.* **69**, 106–114 (1998).

34. Olivieri, G. *et al.* The ever-increasing diversity in mouse lemurs: Three new species in north and northwestern Madagascar. *Mol. Phylogenet. Evol.* **43**, 309–327 (2007).

35. Stankowski, S. & Ravinet, M. Defining the speciation continuum. *Evolution* **75**, 1256–1273 (2021).

36. Schüßler, D. *et al.* Thirty years of deforestation within the entire ranges of nine endangered lemur species in northwestern Madagascar. *Ecotropica* **25**, 202304 (2023).

37. Louis, Jr., E. E. *et al.* Propithecus coquereli. *The IUCN Red List of Threatened Species* vol. e.T18355A1 (2020).

38. Kollman, M. Note sur les genres Chirogale et Microcebus. *Bull. du Museum Natl. d'Histoire Nat.* **16**, 301–304 (1910).

39. Poelstra, J. W. *et al.* RADseq data reveal a lack of admixture in a mouse lemur contact zone contrary to previous microsatellite results. *bioRxiv* 2021.08.12.455854 (2021) doi:10.1101/2021.08.12.455854.

40. Salmona, J. Comparative conservation genetics of several threatened lemur species living in fragmented environments (PhD thesis). (Instituto Gulbenkian de Ciência - ITQB - UNL Oeiras ITQB, 2015).

41. Herrera, J. P. & Dávalos, L. M. Phylogeny and divergence times of lemurs inferred with recent and ancient fossils in the tree. *Syst. Biol.* **65**, 772–791 (2016).

42. Dos Reis, M. *et al.* Using phylogenomic data to explore the effects of relaxed clocks and calibration strategies on divergence time estimation: Primates as a test case. *Syst. Biol.* **67**, 594–615 (2018).

43. Angelis, K. & Dos Reis, M. The impact of ancestral population size and incomplete lineage sorting on Bayesian estimation of species divergence times. *Curr. Zool.* **61**, 874–885 (2015).

44. Edwards, S. & Beerli, P. Perspective: Gene divergence, population divergence, and the variance in coalescence times in phylogeographic studies. *Evolution* **54**, 1839–1854 (2000).

45. Carstens, B. C. & Knowles, L. L. Shifting distributions and speciation: Species divergence during rapid climate change. *Mol. Ecol.* **16**, 619–627 (2007).

46. Tiley, G. P., Poelstra, J. W., dos Reis, M., Yang, Z. & Yoder, A. D. Molecular clocks without rocks: New solutions for old problems. *Trends Genet.* **36**, 845–856 (2020).

47. Leaché, A. D., Harris, R. B., Rannala, B. & Yang, Z. The influence of gene flow on species tree estimation: A simulation study. *Syst. Biol.* **63**, 17–30 (2014).

48. Tseng, S. P., Li, S. H., Hsieh, C. H., Wang, H. Y. & Lin, S. M. Influence of gene flow on divergence dating – implications for the speciation history of *Takydromus* grass lizards. *Mol. Ecol.* **23**, 4770–4784 (2014).

49. Tiley, G. P. *et al.* Estimation of species divergence times in presence of cross-species gene flow. *Syst. Biol.* **72**, 820–836 (2023).

50. Gasse, F. & van Campo, E. Late Quaternary environmental changes from a pollen and diatom record in the southern tropics (Lake Tritrivakely, Madagascar). *Palaeogeogr. Palaeoclimatol. Palaeoecol.* **167**, 287–308 (2001).

51. Tiley, G. P. *et al.* Genetic variation in *Loudetia simplex* supports the presence of ancient grasslands in Madagascar. *Plants, People, Planet* **6**, 315-329 (2023).

52. Everson, K. M., Jansa, S. A., Goodman, S. M. & Olson, L. E. Montane regions shape patterns of diversification in small mammals and reptiles from Madagascar's moist evergreen forest. *J. Biogeogr.* **47**, 2059–2072 (2020).

53. Wilmé, L., Goodman, S. M. & Ganzhorn, J. U. Biogeographic evolution of Madagascar's microendemic biota. *Science* **312**, 1063–1065 (2006).

54. Beck, H. E. *et al.* Present and future Köppen-Geiger climate classification maps at 1 km resolution. *Sci. Data* **5**, 180214 (2018).

55. Goldberg, E. E., Lancaster, L. T. & Ree, R. H. Phylogenetic inference of reciprocal effects between geographic range evolution and diversification. *Syst. Biol.* **60**, 451–465 (2011).

56. Fitzjohn, R. G. Diversitree: Comparative phylogenetic analyses of diversification in R. *Methods Ecol. Evol.* **3**, 1084–1092 (2012).

57. Everson, K. M., Soarimalala, V., Goodman, S. M. & Olson, L. E. Multiple loci and complete taxonomic sampling resolve the phylogeny and biogeographic history of tenrecs (Mammalia: Tenrecidae) and reveal higher speciation rates in Madagascar's humid forests. *Syst. Biol.* **65**, 890–909 (2016).

58. Davis, M. P., Midford, P. E. & Maddison, W. Exploring power and parameter estimation of the BiSSE method for analyzing species diversification. *BMC Evol. Biol.* **13**, 38 (2013).

59. Pennell, M. W., Fitzjohn, R. G., Cornwell, W. K. & Harmon, L. J. Model adequacy and the macroevolution of angiosperm functional traits. *Am. Nat.* **186**, E33–E50 (2015).

60. Felsenstein, J. Phylogenies and quantitative characters. *Annu. Rev. Ecol. Syst.* **19**,

445–471 (1988).

61. Slater, G. J., Harmon, L. J. & Alfaro, M. E. Integrating fossils with molecular phylogenies improves inference of trait evolution. *Evolution* **66**, 3931–3944 (2012).

62. Slater, G. J. & Pennell, M. W. Robust regression and posterior predictive simulation increase power to detect early bursts of trait evolution. *Syst. Biol.* **63**, 293–308 (2014).

63. Lande, R. Natural selection and random genetic drift in phenotypic evolution. *Evolution* **30**, 314–334 (1976).

64. Martins, E. P. & Hansen, T. P. A microevolutionary link between phylogenies and comparative data. in *New Uses for New Phylogenies* (eds. Harvey, P. H., Leigh Brown, A. J., Maynard Smith, J. & Nee, S.) 273–288 (Oxford University Press, 1996).

65. Hansen, T. F. Stabilizing selection and the comparative analysis of adaptation. *Evolution* **51**, 1341–1351 (1997).

66. Zablocki-Thomas, P., Lailvaux, S., Aujard, F., Pouydebat, E. & Herrel, A. Maternal and genetic correlations between morphology and physical performance traits in a small captive primate, *Microcebus murinus*. *Biol. J. Linn. Soc.* **134**, 28–39 (2021).

67. Estes, S. & Arnold, S. J. Resolving the paradox of stasis: Models with stabilizing selection explain evolutionary divergence on all timescales. *Am. Nat.* **169**, 227–244 (2007).

68. Radespiel, U., Lutermann, H., Schmelting, B. & Zimmermann, E. An empirical estimate of the generation time of mouse lemurs. *Am. J. Primatol.* **81**, e23062 (2019).

69. Harmon, L. J. *et al.* Early bursts of body size and shape evolution are rare in comparative data. *Evolution* **64**, 2385–2396 (2010).

70. IUCN. *IUCN Red List categories and criteria*. (2012).

71. Dolch, R., Schüßler, D., Radespiel, U. & M, B. Microcebus lehilahytsara*. The IUCN Red List of Threatened Species*. (2022).

72. Genomic Resources Development Consortium *et al.* Genomic resources notes accepted 1 December 2014 - 31 January 2015. *Mol. Ecol. Resour.* **15**, 684–684 (2015).

73. Etter, P. D., Bassham, S., Hohenlohe, P. A., Johnson, E. A. & Cresko, W. A. SNP discovery and genotyping for evolutionary genetics using RAD sequencing. *Methods Mol. Biol.* **772**, 157–178 (2011).

74. Ali, O. A. *et al.* Rad capture (Rapture): Flexible and efficient sequence-based

genotyping. *Genetics* **202**, 389–400 (2016).

75. Li, H. & Ralph, P. Local PCA shows how the effect of population structure differs along the genome. *Genetics* **211**, 289–304 (2019).

76. Aleixo-Pais, I. *et al.* The genetic structure of a mouse lemur living in a fragmented habitat in northern Madagascar. *Conserv. Genet.* **20**, 229–243 (2019).

77. Jackson, N. D., Carstens, B. C., Morales, A. E. & O'Meara, B. C. Species delimitation with gene flow. *Syst. Biol.* **66**, 799–812 (2017).

78. Wright, P. C. Lemur traits and Madagascar ecology: Coping with an island environment. *Yearb. Phys. Anthropol.* **42**, 31–72 (1999).

79. Radespiel, U., Rakotondravony, R., Rasoloharijaona, S. & Randrianambinina, B. A 24-year record of female reproductive dynamics in two sympatric mouse lemur species in northwestern Madagascar. *Int. J. Primatol.* **43**, 559–583 (2022).

80. Wrogemann, D., Radespiel, U. & Zimmermann, E. Comparison of reproductive characteristics and changes in body weight between captive populations of rufous and gray mouse lemurs. *Int. J. Primatol.* **22**, 91–108 (2001).

81. Dorai-Raj, S. *binom: Binomial confidence intervals for several parameterizations. R package version 1.1*. (2022).

82. Tishechkin, D. Y. The use of bioacoustic characters for distinguishing between cryptic species in insects: Potentials, restrictions, and prospects. *Entomol. Rev.* **94**, 289–309 (2014).

83. Köhler, J. *et al.* The use of bioacoustics in anuran taxonomy: Theory, terminology, methods and recommendations for best practice. *Zootaxa* **4251**, 1–124 (2017).

84. Ramasindrazana, B., Goodman, S. M., Schoeman, M. C. & Appleton, B. Identification of cryptic species of *Miniopterus* bats (Chiroptera: Miniopteridae) from Madagascar and the Comoros using bioacoustics overlaid on molecular genetic and morphological characters. *Biol. J. Linn. Soc.* **104**, 284–302 (2011).

85. Brown, R. M. *et al.* Conservation genetics of the Philippine tarsier: Cryptic genetic variation restructures conservation priorities for an island archipelago primate. *PLoS One* **9**, e104340 (2014).

86. Boersma, P. Praat, a system for doing phonetics by computer. *Glot Int.* **5**, 341–345 (2001).

87. Blonder, B., Lamanna, C., Violle, C. & Enquist, B. J. The *n*-dimensional hypervolume. *Glob. Ecol. Biogeogr.* **23**, 595–609 (2014).

88. Junker, R. R., Kuppler, J., Bathke, A. C., Schreyer, M. L. & Trutschnig, W. Dynamic range boxes – a robust nonparametric approach to quantify size and overlap of *n*-dimensional hypervolumes. *Methods Ecol. Evol.* **7**, 1503–1513 (2016).

89. Dos Reis, M. *et al.* Uncertainty in the timing of origin of animals and the limits of precision in molecular timescales. *Curr. Biol.* **25**, 2939–2950 (2015).

90. Ryan Campbell, C. *et al.* Pedigree-based measurement of the *de novo* mutation rate in the gray mouse lemur reveals a high mutation rate, few mutations in CpG sites, and a weak sex bias. *Heredity* **127**, 233–244 (2021).

91. Jónsson, H. *et al.* Parental influence on human germline *de novo* mutations in 1,548 trios from Iceland. *Nature* **549**, 519–522 (2017).

92. Tatsumoto, S. *et al.* Direct estimation of *de novo* mutation rates in a chimpanzee parent-offspring trio by ultra-deep whole genome sequencing. *Sci. Rep.* **7**, 13561 (2017).

93. Besenbacher, S., Hvilsom, C., Marques-Bonet, T., Mailund, T. & Schierup, M. H. Direct estimation of mutations in great apes reconciles phylogenetic dating. *Nat. Ecol. Evol.* **3**, 286–292 (2019).

94. Pfeifer, S. P. Direct estimate of the spontaneous germ line mutation rate in African green monkeys. *Evolution* **71**, 2858–2870 (2017).

95. Thomas, G. W. C. *et al.* Reproductive longevity predicts mutation rates in primates. *Curr. Biol.* **28**, 3193-3197.e5 (2018).

96. De Manuel, M., Wu, F. L. & Przeworski, M. A paternal bias in germline mutation is widespread in amniotes and can arise independently of cell division numbers. *Elife* **11**, e80008 (2022).

97. Bergeron, L. A. *et al.* The mutationathon highlights the importance of reaching standardization in estimates of pedigree-based germline mutation rates. *Elife* **11**, e73577 (2022).

98. Zohdy, S. *et al.* Teeth, sex, and testosterone: Aging in the world's smallest primate. *PLoS One* **9**, e109528 (2014).
