# Supplementary material for: Integrative taxonomy clarifies the evolution of a cryptic primate clade
Source: Nat Ecol Evol. 2024 Sep 27;9(1):57–72. doi: 10.1038/s41559-024-02547-w (PMC11726463; doi:10.1038/s41559-024-02547-w)
Supplement: Supplementary file 2 — Reporting Summary [file 41559_2024_2547_MOESM2_ESM.pdf]

nature portfolio

Corresponding author(s): Jordi Salmona, Tobias van Elst

Last updated by author(s): Aug 9, 2024

Reporting Summary

Nature Portfolio wishes to improve the reproducibility of the work that we publish. This form provides structure for consistency and transparency in reporting. For further information on Nature Portfolio policies, see our Editorial Policies and the Editorial Policy Checklist.

Statistics

For all statistical analyses, confirm that the following items are present in the figure legend, table legend, main text, or Methods section.

n/a	Confirmed	
☐	☒	The exact sample size (*n*) for each experimental group/condition, given as a discrete number and unit of measurement
☐	☒	A statement on whether measurements were taken from distinct samples or whether the same sample was measured repeatedly
☐	☒	The statistical test(s) used AND whether they are one- or two-sided<br>*Only common tests should be described solely by name; describe more complex techniques in the Methods section.*
☐	☒	A description of all covariates tested
☐	☒	A description of any assumptions or corrections, such as tests of normality and adjustment for multiple comparisons
☐	☒	A full description of the statistical parameters including central tendency (e.g. means) or other basic estimates (e.g. regression coefficient) AND variation (e.g. standard deviation) or associated estimates of uncertainty (e.g. confidence intervals)
☐	☒	For null hypothesis testing, the test statistic (e.g. *F*, *t*, *r*) with confidence intervals, effect sizes, degrees of freedom and *P* value noted<br>*Give P values as exact values whenever suitable.*
☐	☒	For Bayesian analysis, information on the choice of priors and Markov chain Monte Carlo settings
☒	☐	For hierarchical and complex designs, identification of the appropriate level for tests and full reporting of outcomes
☐	☒	Estimates of effect sizes (e.g. Cohen's *d*, Pearson's *r*), indicating how they were calculated

Our web collection on statistics for biologists contains articles on many of the points above.

Software and code

Policy information about availability of computer code

Data collection	No software was used for data collection.
Data analysis	RAD genotyping: Stacks v2.0b, Trimmomatic v0.39, BWA-MEM v0.7.17, SAMtools v1.11, GATK v4.1.9.0, VCFtools v0.1.17, MUSCLE v3.8.31, ANGSD v0.92<br>Phylogenetic inference: IQ-TREE v2.2.0, PAUP* v4.0a<br>Species delimitation: NGSadmix v32, PRAAT v5.4.0.4, R packages 'lostruct' v0.0.0.9000, 'dynRB' v0.18, 'ENMtools' v1.0.7, 'ENMeval' v2.0, 'binom' v1.1-1.1<br>Divergence time estimation: BPP v4.4.1, Tracer v1.7.2<br>Biogeographic reconstruction: R package 'BioGeoBears' v1.1.2<br>Modelling morphological and climatic niche evolution: R packages 'phytools' v2.3-0, 'dynRB' v0.18, 'phyloclim' v0.9.5, 'mvMORPH' v1.1.9, 'tmvtnorm' v1.6<br>Conservation reassessment: ArcGIS Pro v3.1.0<br>All custom analysis scripts can be found at https://github.com/t-vane/van_Elst_et_al_2024_Cryptic_diversification.

For manuscripts utilizing custom algorithms or software that are central to the research but not yet described in published literature, software must be made available to editors and reviewers. We strongly encourage code deposition in a community repository (e.g. GitHub). See the Nature Portfolio guidelines for submitting code & software for further information.

Data

Policy information about availability of data

All manuscripts must include a data availability statement. This statement should provide the following information, where applicable:

- Accession codes, unique identifiers, or web links for publicly available datasets
- A description of any restrictions on data availability
- For clinical datasets or third party data, please ensure that the statement adheres to our policy

All new sequencing data have been made available through NCBI BioProjects PRJNA560399 and PRJNA807164. Individual BioSample accessions are given in Supplementary Table 13.
Bioclimatic data were extracted from CHELSEA database v2.1 (https://envicloud.wsl.ch/#/?prefix=chelsa%2Fchelsa_V1%2Fclimatologies).
Occurrence records are given in Supplementary Table 15.
Reproductive data are given in Supplementary Table 16.
Morphometric and acoustic raw data as well as all analysis input, output and configuration files are available at Dryad (https://doi.org/10.5061/dryad.b2rbnzsp3).

Research involving human participants, their data, or biological material

Policy information about studies with human participants or human data. See also policy information about sex, gender (identity/presentation), and sexual orientation and race, ethnicity and racism.

Reporting on sex and gender	Not applicable.
Reporting on race, ethnicity, or other socially relevant groupings	Not applicable.
Population characteristics	Not applicable.
Recruitment	Not applicable.
Ethics oversight	Not applicable.

Note that full information on the approval of the study protocol must also be provided in the manuscript.

Field-specific reporting

Please select the one below that is the best fit for your research. If you are not sure, read the appropriate sections before making your selection.

☐ Life sciences ☐ Behavioural & social sciences ☒ Ecological, evolutionary & environmental sciences

For a reference copy of the document with all sections, see nature.com/documents/nr-reporting-summary-flat.pdf

Ecological, evolutionary & environmental sciences study design

All studies must disclose on these points even when the disclosure is negative.

Study description	We developed a practical integrative framework following that considers genomic, bioclimatic, morphometric, and behavioural data to consistently delimit species across taxonomically challenging groups. We applied this framework to the genus Microcebus, including all 25 named species with extensive geographic sampling. We inferred the temporal evolution of habitat and climatic niche, and their combined impacts on morphological stasis through time. Additionally, we highlighted the consequences for conservation status and identified phylogeographic conservation units.
Research sample	The genus Microcebus was chosen as model to illustrate the presented integrative approach to taxonomy and diversification research due to its cryptic diversity, controversial taxonomy and relative wealth of available data. We compiled all available information across multiples lines of evidence (genomics, morphometrics, bioclimate, reproduction, accoustic communication) for the genus from the literature and our own research. Accordingly, the research sample aimed to be as comprehensive as possible, covering all described and one putative Microcebus species.
Sampling strategy	Sampling aimed to be comprehensive accounting for the full geno- and phenotypic diversity known in the genus Microcebus. It therefore covered all described and assumed distributions of Microcebus species across Madagascar.
Data collection	Microcebus samples were obtained by numerous researchers (given in Supplementary Tables 13 to 17). This included taking tissues (i.e.,ear biopsies) for genomic analyses, morphometric measurements, bioclimatic data, sex, and data on acoustic communication. Sampling procedures for each of these are given in the Methods in the manuscript.
Timing and spatial scale	Microcebus individuals were sampled between 1994 and 2022 across all forested ecosystems of Madagascar. Sampling localities and dates are given in Tables 13 to 17.

Data exclusions

For genomic analyses, several commonly used filters were applied on both individual and genotype level to limit bias from data quality (sequencing depth and quality; described in Methods). Morphometric data were filtered following Schüßler et al. (2023, AJBA; see Methods). Bioclimatic data was filtered on individual level to reduce autocorrelation.

Reproducibility

All scripts, input, output and configuration files as well as intermediate results are made available through online repositories, promoting full reproducibility of our study.

Randomization

Samples were not randomized as no manipulative experiments were carried out. Individuals were assigned to candidate species based on geographic location, preliminary identification of the respective field primatologist and on previous sequencing activites in different laboratories.

Blinding

Not applicable.

Did the study involve field work? ☒ Yes ☐ No

Field work, collection and transport

Field conditions

Field work was carried out over the course of approx. 30 years during both dry and rainy season. Detailed sampling dates are given in Supplementary Tables 13 to 17.

Location

Field work was carried out in forested ecosystems across Madagascar. Detailed sampling locations are given in Supplementary Tables 13 to 17.

Access & import/export

Research was conduted with the permission of the following authorities and institutions: The Direction Générale du Ministère de l'Environnement et des Forêts de Madagascar, and Madagascar's Ad Hoc Committee for Fauna and Flora and Organisational Committee for Environmental Research (CAFF/CORE). Associated permit numbers are:
[87,233,329]/06/10/MEF/SG/DCB.SAP/SCB
121/07/MEF/SG/DCB.SAP/SCB
[026,227]/08/10/MEF/SG/DCB.SAP/SCB
[218,220]/10/MEF/SG/DCB.SAP/SCB
[113,118,186]/11/MEF/SG/DCB.SAP/SCB
[074,099,124]/12/MEF/SG/DCB.SAP/SCB
[137,175,178,179,186]/13/MEF/SG/DCB.SAP/SCB
[150,168,169,137]/13/MEF/SG/DGF/DCB.SAP/SCB
175/14/MEF/SG/DCB.SAP/SCB
072/15/MEEMF/SG/DGF/DCB.SAP/SCB
074/15/MEEEMEF/SG/DGF/DCB.SAP/SCD
[169,270]/16/MEEF/SG/DGF/DSAP/SCB.Re
130/16/MEEF/SG/DGF/DAPT/SCBT.Re
[044,078,079,136,197]/17/MEEF/SG/DGF/DSAP/SCB.Re
[080,151]/17/MEF/SG/DGF/DSAP/SCB.Rc
[035,084,093]/18/MEF/ SG/DGF/DSAP/SCB.Rc.
013/19/MEEF/SG/DGF/DSAP/SCB.Re
169/19/MEDD/SG/DGEF/DGRNE
349/21/MEDD/SG/DGGE/DAPRNE/SCBE.Re
[015,030]/22/MEDD/SG/DGGE/DAPRNE/SCBE.Re
[023,037,071,072,181]/MINENV.EF/SG/DGEF/DADF/SCB
[093,179,230]/MINENV.EF/SG/DGEF/DPB/SCBLF
[075,177]/MINENV.EF/SG/DGEF/DPB/SCBLF/RECH
[87,233,329]/06/10/MEF/SG/DCB.SAP/SCB
121/07/MEF/SG/DCB.SAP/SCB
[026,227]/08/10/MEF/SG/DCB.SAP/SCB

Disturbance

Disturbance was minimized by releasing captured Microcebus individuals within 24 hours of capture.

Reporting for specific materials, systems and methods

We require information from authors about some types of materials, experimental systems and methods used in many studies. Here, indicate whether each material, system or method listed is relevant to your study. If you are not sure if a list item applies to your research, read the appropriate section before selecting a response.

Materials & experimental systems

n/a	Involved in the study
☒	☐ Antibodies
☒	☐ Eukaryotic cell lines
☒	☐ Palaeontology and archaeology
☐	☒ Animals and other organisms
☒	☐ Clinical data
☒	☐ Dual use research of concern
☒	☐ Plants

Methods

n/a	Involved in the study
☒	☐ ChIP-seq
☒	☐ Flow cytometry
☒	☐ MRI-based neuroimaging

Animals and other research organisms

Policy information about studies involving animals; ARRIVE guidelines recommended for reporting animal research, and Sex and Gender in Research

Laboratory animals	No laboratory animals were used in this study.
Wild animals	Wild mouse lemurs were captured during night by hand or with Sherman traps baited with banana. Capture procedures are reported and referenced in the Methods of the manuscript. After transport to the camping site in Sherman traps, Microcebus individuals were measured (morphometrics) and tissue samples (i.e., ear biopsies) were taken. Individuals were released within 24 hours of capture at the respective capture location. Species and/or developmental stage of sampled mouse lemurs are provided in Supplementary Tables 13 to 17.
Reporting on sex	Sex of animals was only relevant to the analysis of reproductive activity and is reported for all sampled animals in Supplementary Tables 13 to 16.
Field-collected samples	Tissue samples were stored in Queen's lysis buffer or 70% ethanol. They were preserved at room temperature during the field season and were frozen at -20°C afterwards and until DNA extraction.
Ethics oversight	This study was conducted in agreement with the laws of Madagascar and adhered to the principles of the Code of Best Practices for Field Primatology of the International Primatological Society and the ethical guidelines of the Association for the Study of Animal Behaviour and the Animal Behavior Society. All capture and handling procedures followed routine protocols and were approved by the Malagasy Authorities.

Note that full information on the approval of the study protocol must also be provided in the manuscript.

Plants

Seed stocks	Not applicable.
Novel plant genotypes	Not applicable.
Authentication	Not applicable.
